# Evolutionary diversification of the RomR protein of the invasive deltaproteobacterium, *Bdellovibrio bacteriovorus*

**DOI:** 10.1038/s41598-019-41263-5

**Published:** 2019-03-21

**Authors:** Rebecca C. Lowry, David S. Milner, Asmaa M. S. Al-Bayati, Carey Lambert, Vanessa I. Francis, Steven L. Porter, R. E. Sockett

**Affiliations:** 1 School of Life Sciences, University of Nottingham, Medical School, Nottingham, United Kingdom; 2 Biosciences, College of Life and Environmental Sciences, University of Exeter, Exeter, United Kingdom; 3 Northern Technical University, Mosul, Iraq

## Abstract

*Bdellovibrio bacteriovorus* is a predatory deltaproteobacterium that encounters individual Gram-negative prey bacteria with gliding or swimming motility, and then is able to invade such prey cells via type IVa pilus-dependent mechanisms. Movement control (pili or gliding) in other deltaproteobacteria, such as the pack hunting *Myxococcus xanthus*, uses a response regulator protein, RomR_Mx_ (which dynamically relocalises between the cell poles) and a GTPase, MglA_Mx_, previously postulated as an interface between the Frz_Mx_ chemosensory system and gliding or pilus-motility apparatus, to produce regulated bidirectional motility. In contrast, *B. bacteriovorus* predation is a more singular encounter between a lone predator and prey; contact is always via the piliated, non-flagellar pole of the predator, involving MglA_Bd_, but no Frz system. In this new study, tracking fluorescent RomR_Bd_ microscopically during predatory growth shows that it does not dynamically relocalise, in contrast to the *M. xanthus* protein; instead having possible roles in growth events. Furthermore, transcriptional start analysis, site-directed mutagenesis and bacterial two-hybrid interaction studies, indicate an evolutionary loss of RomR_Bd_ activation (via receiver domain phosphorylation) in this lone hunting bacterium, demonstrating divergence from its bipolar role in motility in pack-hunting *M. xanthus* and further evolution that may differentiate lone from pack predators.

## Introduction

The predatory deltaproteobacterium, *Bdellovibrio bacteriovorus*, can invade and replicate intraperiplasmically within other Gram-negative bacteria. During the host-dependent (HD) life cycle, *B. bacteriovorus* begins in a non-replicating state, known as attack-phase, where cells are hunting for regions rich in prey and are ready to invade them. Prey encounters occur randomly due to predator gliding motility on solid surfaces^1^, or rapid swimming motility (speeds up to 160 µm/s have been observed) via a single polar flagellum in liquid cultures^2^. The opposite cell pole to the flagellum-containing pole is the prey-invasive pole, responsible for predator attachment to prey and contains type IVa pili which are crucial for the invasion process^3,4^. Defined cell poles are essential in the “attack-phase” (non-saprophytically replicating) state of predators for efficient prey attachment and entry in the predatory lifestyle. *B. bacteriovorus* strains can, as an alternative, switch to grow host/prey-independently (HI), a process requiring point mutations in *Bdellovibrio*-specific genes to occur. This HI lifestyle can be utilised experimentally to save, culture and analyse non-predatory mutants such as type IVa pilus-defective strains that are incapable of replicating predatorily^3^.

We previously reported there to be a regulatory ‘protein hub’ at the prey-invasive pole of *B. bacteriovorus*, with a number of the proteins involved having roles in the predatory process and beyond^5^. Intriguingly, homologues to proteins that regulate directed motility in the closely related *Myxococcus* (RomR and MglA) are members of this regulatory hub, but divergent evolution has given MglA_Bd_ an alternative role in *B. bacteriovorus*^5^.

In *Myxococcus xanthus*, the RomR/MglA/MglB system establishes the cell polarity required for bidirectional motility to occur (social and adventurous) in response to the Frz chemosensory system^6^. In brief, the Ras-like GTPase, MglA, stimulates the assembly of motility apparatus at the leading cell pole when GTP-bound. The MglA-specific GTPase activating protein (GAP), MglB, is predominantly present at the lagging cell pole, preventing the accumulation of GTP bound MglA. It is the asymmetric localisation of MglA and MglB that controls cell polarity, and the Frz chemosensory system is responsible for the frequency of *M. xanthus* cell reversals^6^ by inducing the relocalisation of MglA to the opposite pole, which switches the leading and lagging poles (enabling motility reversals)^7,8^. The link between the MglA/MglB polarity proteins and the Frz system, in *M. xanthus*, was thought to be the response regulator protein, RomR_Mx_, which has been observed to dynamically relocalise between the poles and has affinity for GTP-bound MglA_Mx_, enabling redistribution of the proteins at the cell poles allowing motility reversals to occur^6,9–11^. Furthermore, previous mutational studies on the RomR_Mx_ REC domain (conserved, putative phosphate-accepting residue, aspartate 53) have suggested the protein to be phosphorylated by the Frz system, therefore inducing the dynamic relocalisation events^9^. Nevertheless, a RomR_Mx_-kinase has never been identified, despite the response regulator-kinase hybrid, FrzE, being a good candidate as it acts upstream of RomR_Mx_ in the Frz-polarity switching hierarchy and its mutation/deletion has a severe impact on cell reversal frequency^12,13^. Recent work by Guzzo *et al*.^12^ contradicts these findings, however, and demonstrates that RomR_Mx_ is not phosphorylated by FrzE nor is its phosphorylation site required for motor reversals. Their findings establish that RomR_Mx_ is not the only link between the Frz system and the polarity proteins, and that a newly identified response regulator protein, FrzX, works together with RomR_Mx_ and is key for triggering cell reversals upon direct phosphorylation by FrzE^12^. RomR_Mx_ was found to prime the cell for the next reversal, with its movement to the opposite pole acting as a refractory period preventing activation of cell reversals immediately after one had taken place^12^.

Although MglA and RomR homologues are present in *B. bacteriovorus*, MglB is absent, and fragments (only) of the *mglB* gene can be observed within the *B. bacteriovorus* HD100 genome; again implying divergence from the *Myxococcus* role^5,14^. Furthermore, work by Milner *et al*.^5^ demonstrated that a *B. bacteriovorus mglA* deletion strain is unable to carry out the HD-life cycle, suggesting this protein may be involved in controlling prey-invasion regulation rather than motility. Initial studies on the RomR homologue (RomR_Bd_) were surprising, however, as *romR*_*Bd*_ was found to be essential for viability, implying a wider role for this protein in *B. bacteriovorus* survival than just the predatory process^5^. Furthermore, the *frz* genes are not present within the *B. bacteriovorus* genome and so whether RomR_Bd_ is responding to signals from another system to carry out a distinct role in this intraperiplasmic predator remains unknown.

In this study, we report that expression of *B. bacteriovorus* RomR_Bd_ increases during intracellular growth of this predator, a life cycle period where there is no significant movement and no requirement for dynamic relocalisation. Site-directed mutagenesis, transcriptomic and RT-PCR studies conclude that RomR_Bd_ has a truncated REC domain, providing evidence that this protein is not regulated by phosphorylation. We identify a pseudogene encoding a potential cognate sensor kinase for RomR_Bd_, Bd2406, which has a stop codon upstream of its kinase region, indicating that the ability to phosphorylate RomR_Bd_ has been lost. These data suggest that this system has diverged from the motility role observed in the deltaproteobacterial relative, *M. xanthus*.

## Results

### RomR_Bd_ bioinformatic analysis and localisation throughout the *B. bacteriovorus* HD100 cell cycle

**Figure 1:**
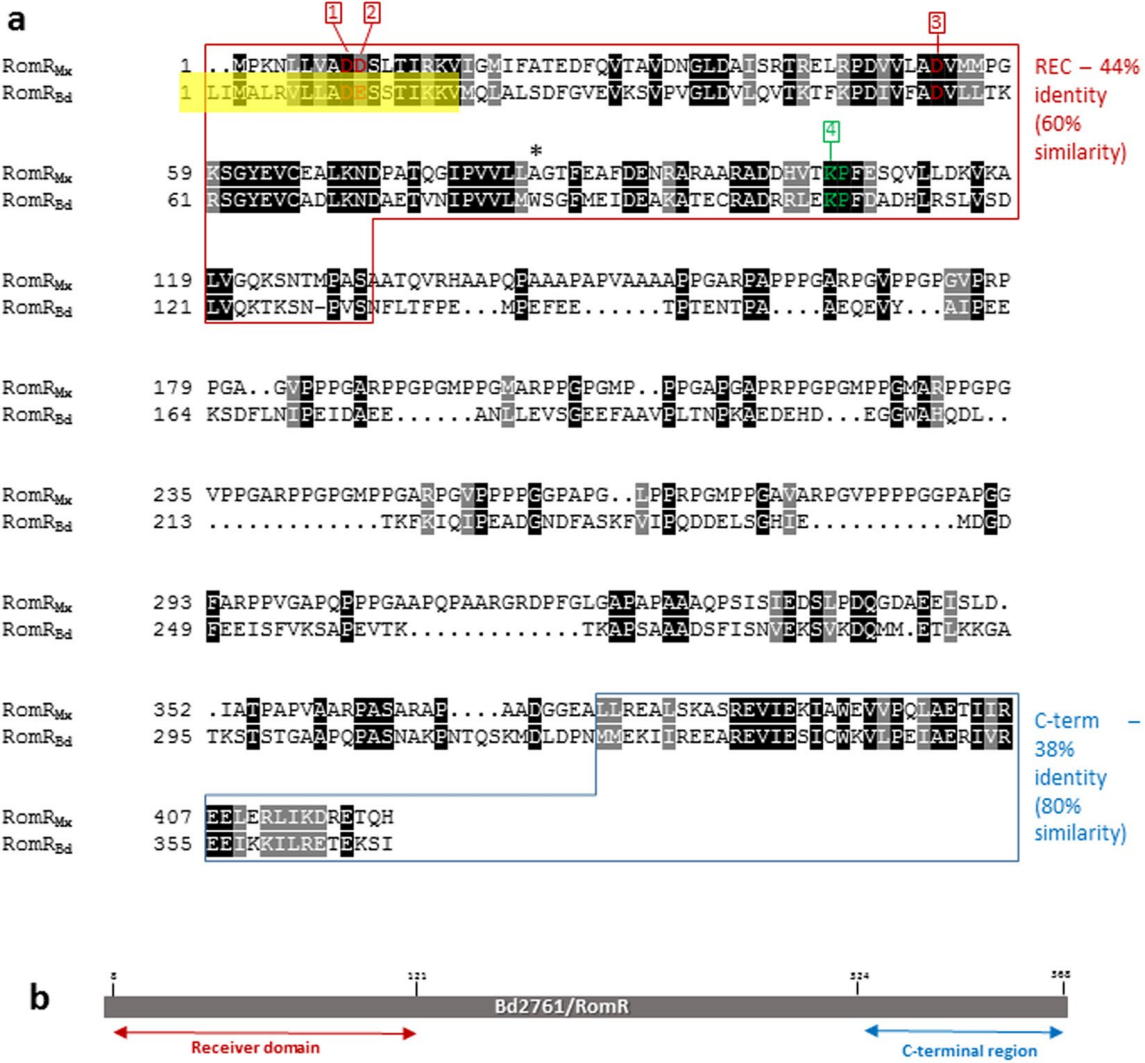
(**a**) Alignment of the initially predicted *B. bacteriovorus* HD100 RomR (RomR_Bd_) amino acid sequence^14^ with the *M. xanthus* DK 1622 RomR (RomR_Mx_) sequence^41^. Alignment carried out using CLUSTALW and displayed with BoxShade. The N-terminal receiver domains (REC) share 44% identity and residues important for a fully functional REC domain are highlighted. Positions 1, 2 and 3 highlight the predicated acidic triad, likely important for Mg^2+^ coordination required for phosphorylation, with D55 of RomR_Bd_ (or D53 of RomR_Mx_) at position 3, being the predicted sites of phosphorylation within this triad^15,16^. A conserved lysine residue (at position 107 in the HD100 protein) is also highlighted (*), this is likely important for Mg^2+^ coordination. The C-terminal regions of RomR_Bd_ and RomR_Mx_ are also conserved, displaying 38% identity. The yellow box over the RomR_Bd_ sequence shows the 19 amino acids of the REC domain, which we go on to show are not expressed in *B. bacteriovorus* proteins. (**b**) Diagrammatic representation of the position of the predicted domains of the *B. bacteriovorus* RomR on the unfolded protein.

Analysis of the *B. bacteriovorus romR* gene (*romR*_*Bd*_) was firstly carried out from the genome sequence published by Rendulic and co-workers^14^. The annotation of this genome predicted the *B. bacteriovorus* HD100 gene product, RomR_Bd_ (encoded by *bd2761*) to consist of an N-terminal receiver domain (REC), which shares 44% protein sequence identity to the *M. xanthus* homologue (RomR_Mx_) REC domain, and a C-terminal region that is 38% identical to the comparable region of RomR_Mx_; the central region is non-conserved^5^ (Fig. 1a,b). Within the HD100 RomR_Bd_ REC domain, residues required for producing an acidic pocket and for phosphoryl group acceptance were present in the annotation (Fig. 1a). These include the Mg^2+^ co-ordinating residues D_11_E_12_ and K_107_ (Fig. 1a), which, in active REC domains, form salt bridges with the phosphoryl group^15,16^. In addition, there was a predicted phosphorylation site (aspartate 55) that aligned with the conserved site, aspartate 53, in the *M. xanthus* RomR_Mx_^9^. This initially seemed consistent with the RomR_Bd_ REC domain being functional and phosphorylatable (Fig. 1a,b).

RomR_Bd_ was found to interact with a protein complex positioned at the predatory-pole of attack-phase *B. bacteriovorus* HD100^5^, however its fate throughout the predatory life cycle was previously unknown. The *M. xanthus* RomR_Mx_ dynamically relocalises between the poles^9^; this is key to establishing polarity for bidirectional motility in *Myxococcus*^10,11^. To investigate the movements of *B. bacteriovorus* HD100 RomR_Bd_ during the prey invasion and replication process, the protein was tagged C-terminally with the fluorescent protein mCherry and localisation observed during synchronous infection of *E. coli* prey over four hours.

**Figure 2:**
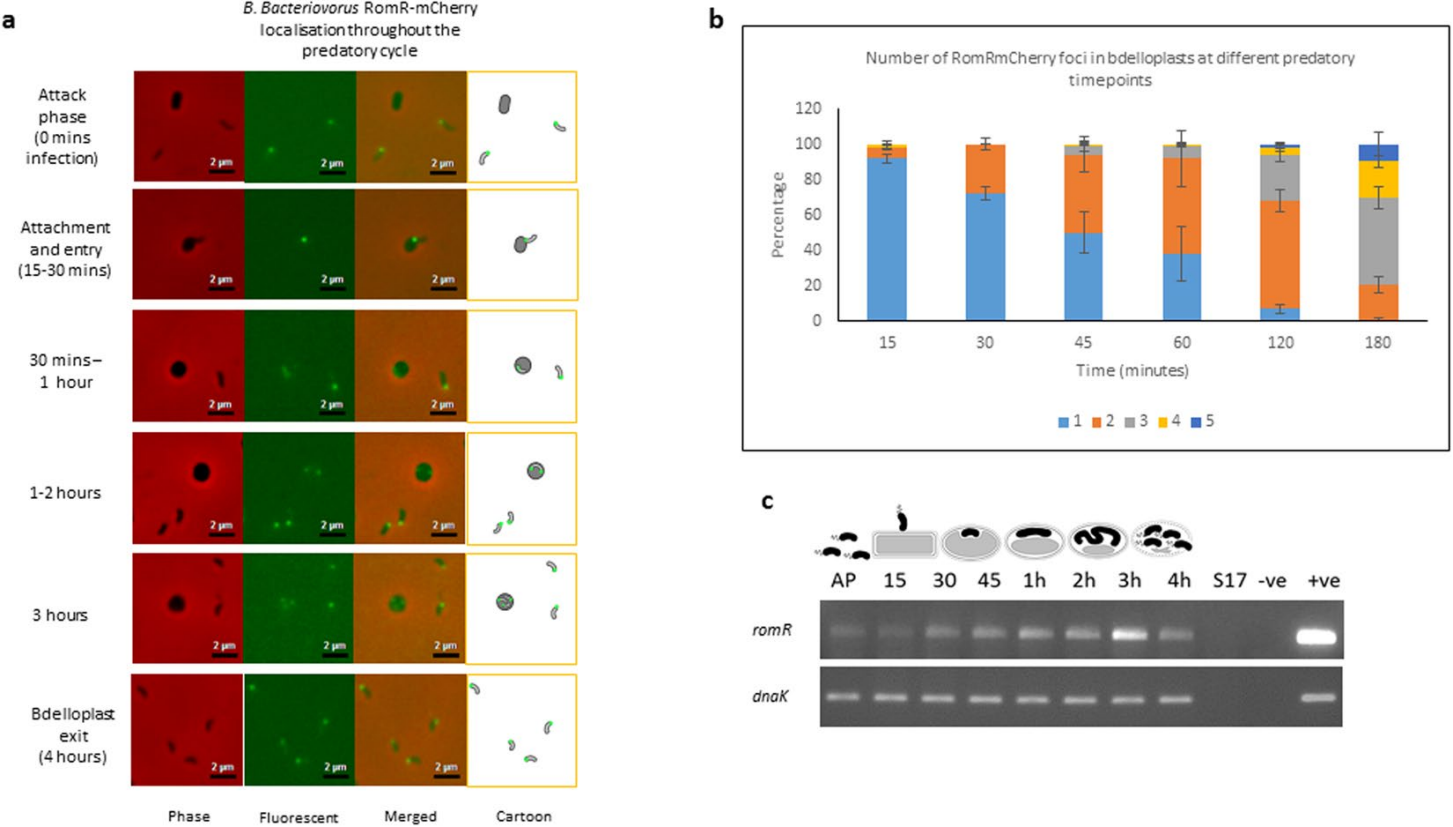
(**a**) Epifluorescence and phase contrast microscopy displaying the localisation of *B. bacteriovorus* RomR tagged C-terminally with mCherry, during invasion and replication within *E. coli* prey (predatory life cycle between 0–4 hours). Images are representative of data collected from three biological replicates. Some free, non-replicating *B. bacteriovorus* with monopolar RomR are also observed outside prey. (**b**) Stacked bar chart showing the distribution of the number of fluorescent RomRmCherry foci per bdelloplast throughout the predatory timecourse. Images from three independent experiments were analysed and bdelloplasts scored for the number of fluorescent foci visible. The mean number of foci observed per bdelloplast increased significantly throughout the timecourse (*p* < 0.0001 by one way ANOVA). Error bars are SEM. Values of n are shown in Supplementary Table S3. (**c**) Expression pattern of *B. bacteriovorus* HD100 *romR* and control gene *dnaK* throughout the predatory life cycle using RT-PCR with transcript specific primers. RNA was prepared from identical volumes of infection cultures with *B. bacteriovorus* HD100 predator and *E. coli* S17-1 prey at time-points throughout the predatory life cycle. AP: RNA from attack-phase *B. bacteriovorus*, 15–45: RNA from 15–45 minute time-points through the predatory cycle, 1–4 h: RNA from 1–4 hours through the time-course, S17: *E. coli* S17-1 only template RNA, −ve: no template negative control, +ve: *B. bacteriovorus* HD100 genomic DNA template as positive control. (Full length agarose gel is presented in Supplementary Fig. S9).

As previously suggested by its interactions with polar proteins^5^ RomR_Bd_-mCherry is found to be located at the attacking (prey contacting) pole of *B. bacteriovorus* in attack-phase (Fig. 2a). Shortly after prey entry (~30 mins) the monopolar RomR_Bd_-mCherry begins to diffuse though the cytoplasm of the *Bdellovibrio* cell (Fig. 2a,b), and then adopts a bipolar position within the intra-bacterial predator. As *B. bacteriovorus* elongates (between 1–2 hours). By 3 hours, multiple foci can be observed within the bdelloplast (Fig. 2a,b) which are likely the predatory poles of each of the new progeny predators forming along the growing filament, and by 4 hours (Fig. 2a) prey lysis has occurred and the progeny are released, with RomR_Bd_-mCherry once again being located at the prey-attacking, non-flagellate pole (Fig. 2a). The average number of RomR mcherry foci increased significantly (*p* < 0.0001 by one-way ANOVA) from 1.1 ± 0.11 at the 15 minute timepoint to 3.19 ± 0.39 at the 3 hour timepoint (Fig. 2b).

Analysis of *romR*_*Bd*_ transcription timing (with primers amplifying DNA region 700–800 bp within the predicted gene sequence) also echoed findings from the fluorescent tagging studies and demonstrated expression throughout the prey-internal life cycle, with increased expression between 1–3 hours during the filamentous growth period of *Bdellovibrio* (Fig. 2c). Expression peaks at 3 hours, which is around the time of septal formation, whereas a control gene *dnaK* is expressed at a constant level throughout the timecourse (Fig. 2c).

**Figure 3:**
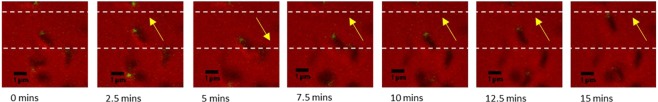
Epifluorescence time-lapse microscopy over a 15 minute period displaying constant localisation of RomR-mCherry during *B. bacteriovorus* gliding motility. The strain, *B. bacteriovorus* RomR-mCherry, was incubated on a 1% agarose surface for 2 hours prior to time-lapse microscopy in order to allow gliding motility to initiate. The RomR-mCherry focus stayed at one pole regardless of direction of motility. Yellow arrow shows direction of motility. Images are representative of 149 cell reversals observed in two independent experiments. Further examples are displayed in Supplementary Fig. S1.

RomR_Bd_-mCherry displayed a fixed cellular location during *B. bacteriovorus* HD100 surface gliding motility, at the leading cell pole. Only small gliding reversals were observed during this surface motility, allowing *B. bacteriovorus* cells to briefly change direction; cells then continued to lead with their predatory (RomR_Bd_-mCherry-containing) poles (Fig. 3; 149 individual cell reversals observed). The fluorescent foci were fixed at the same pole during these small reversals and so were actually at the lagging pole during reversal; this same pole then reverted to being the leading pole as motility reverted to the direction initially observed before the brief, stuttering, reversal. This indicates that RomR_Bd_-mCherry is permanently located at the piliated, non-flagellated attacking pole^5^ during attack phase, and that this is the predominant leading pole for gliding motility.

### Site-directed mutagenesis studies of the RomR_Bd_ REC domain

Previously Milner and coworkers^5^ demonstrated that *romR* could not be deleted from the *B. bacteriovorus* HD100 genome in either host-dependent or -independent lifestyles, suggesting that RomR_Bd_ is essential for survival. Prior studies on the REC domain of the *M. xanthus* homologue have suggested that RomR_Mx_ is phosphorylated on aspartate 53, as mutagenesis of this site had significant effects on gliding reversal frequencies^9^, although recent work by Guzzo *et al*.^12^ contradicts this view. Site-directed mutagenesis studies at the conserved putative phosphorylation site on *B. bacteriovorus* HD100 RomR_Bd_ (aspartate 55) were carried out to test the role of this protein and its phosphorylation status. Two site-directed mutant strains were created, one where RomR_Bd_ D55 was mutated to an alanine residue (D55A), creating a strain where RomR_Bd_ can no longer be phosphorylated. The other mutation was to a glutamate residue (D55E); this has been shown to behave in some circumstances as a phosphomimic^9,17^ and so potentially producing a protein that behaves as if permanently phosphorylated. The wild type *romR*_*Bd*_ gene was successfully replaced on the *B. bacteriovorus* HD100 genome with the mutant versions. Each point mutant was viable and able to carry out the predatory life cycle within 4–5 hours, similar to the wild type strain, which completes the predatory cycle in 4 hours.

**Figure 4:**
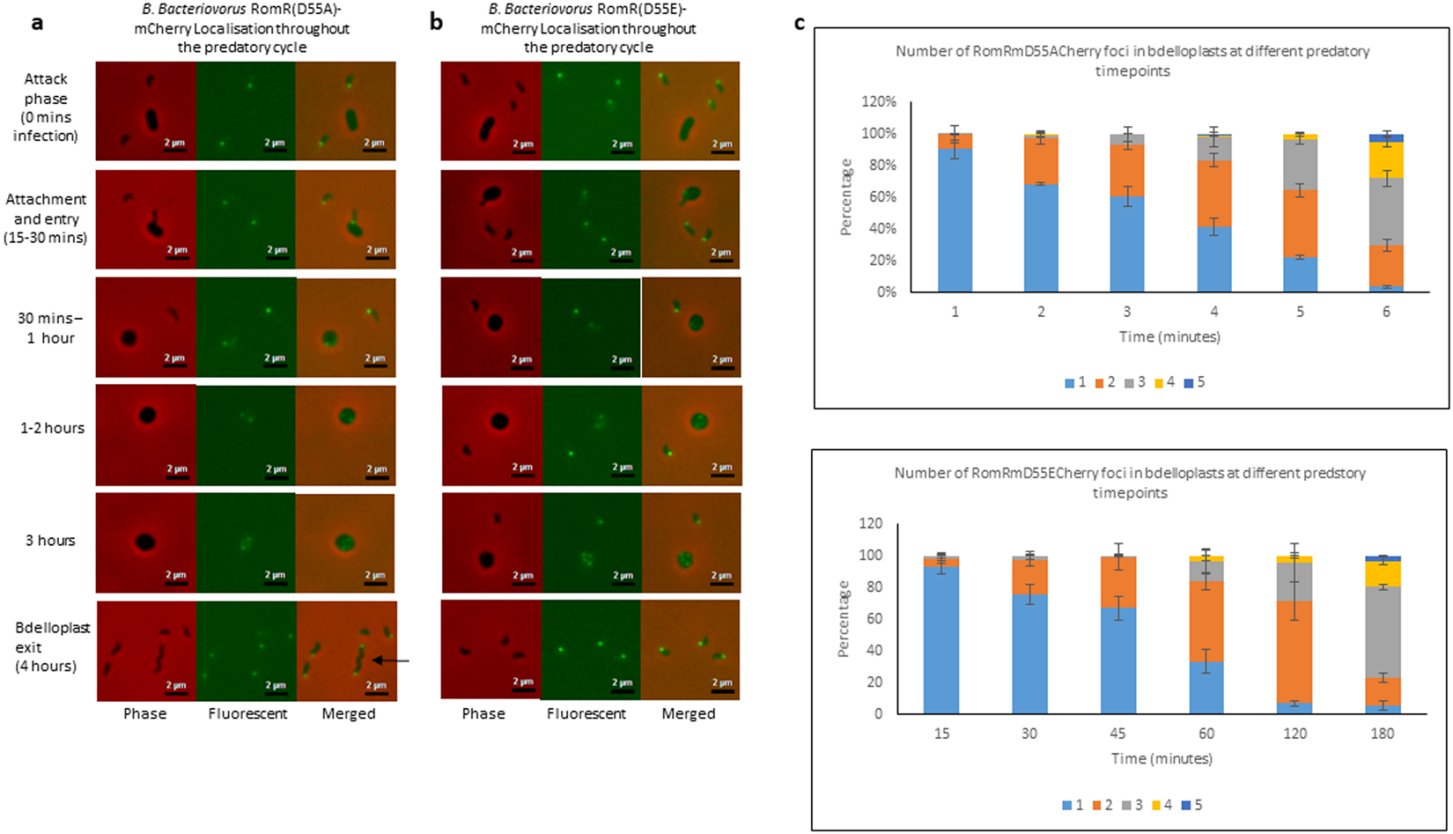
Epifluorescence phase contrast microscopy displaying the localisation of the site-directed mutant *B. bacteriovorus* RomR proteins: RomR(D55A) (**a**) and RomR(D55E) (**b**). The proteins were tagged C-terminally with mCherry in order to visualise RomR localisation during *B. bacteriovorus* invasion and replication within *E. coli* prey (predatory life cycle between 0–4 hours). Images are representative of data collected from three biological replicates. Some longer cells were observed (arrow) in the D55A mutant. Scale bars are 2 µm. (**c**) Stacked bar chart showing the number of fluorescent RomRmCherry foci per bdelloplast throughout the predatory timecourse for mutant (D55A or D55E) RomR. Images from three independent experiments were analysed and bdelloplasts scored for the number of fluorescent foci visible. The mean number of foci observed per bdelloplast increased significantly throughout the timecourse for both strains (*p* < 0.0001 by one way ANOVA). Error bars are SEM. Values of n are shown in Supplementary Table S3.

The site-directed mutant RomR_Bd_ proteins were then tagged C-terminally with mCherry in order to visualise their positions throughout the predatory cycle; these were expressed from genes integrated as single cross-overs from the suicide plasmid pK18*mobsacB* in the genomes of the corresponding site-directed mutant strains (to ensure the mutant proteins were the sole type of RomR in those strains) (Fig. 4). Both mutant versions of RomR, RomR(D55A)mCherry and RomR(D55E)mCherry, localised in a comparable manner to the wild type protein when tagged with mCherry, with significantly increasing numbers of fluorescent foci throughout the timecourse (*p* < 0.0001 by one way ANOVA for each strain; Fig. 4). The mean numbers of foci at each timepoint were not significantly different to those of the wild-type RomRmCherry (*p* = 0.92 for 15 mins, *p* = 0.52 for 30 mins, *p* = 0.38 for 45 mins, *p* = 0.82 for 60 mins, *p* = 0.58 for 120 mins and *p* = 0.50 for 180 mins by one way ANOVA). These data do not suggest a strong role for RomR phosphorylation affecting polar positioning or activity of RomR in *B. bacteriovorus* HD100.

**Figure 5:**
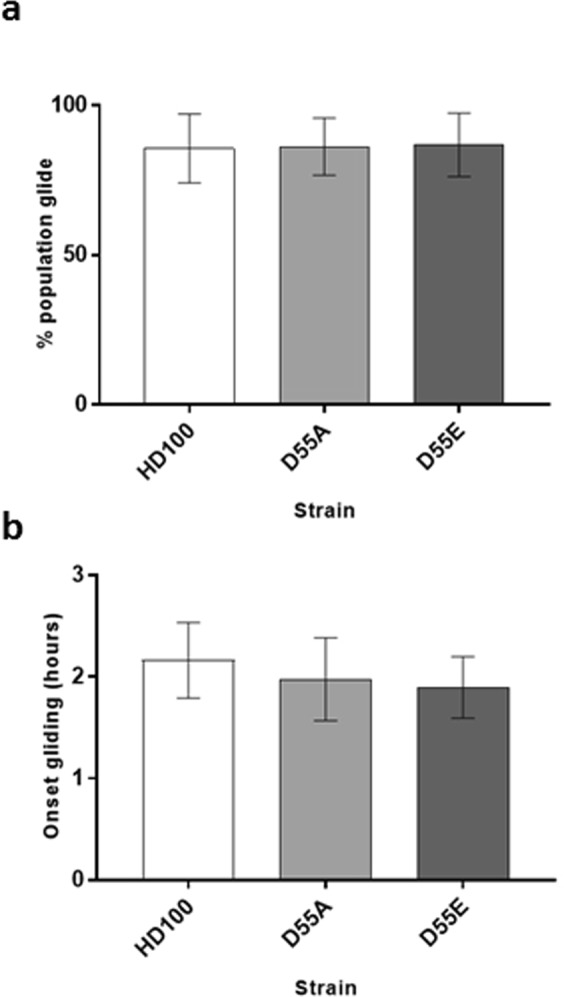
Gliding ability of wild type *B. bacteriovorus* HD100 (n = 1416) compared to the site-directed mutant RomR strains: *B. bacteriovorus* RomR(D55A) [D55A] (n = 1476) and *B. bacteriovorus* RomR(D55E) [D55E] (n = 1736). (**a**) Time-lapse microscopy was carried out over 6 hours, on a 1% agarose surface, to investigate the percentage of each *B. bacteriovorus* population that was able to glide. Between 69–94% (av. 86.2%) of the D55A and 70–95% (av. 86.8%) of the D55E site-directed mutant strain populations analysed were able to glide and this was not significantly different from the gliding ability of the wild type strain where between 69–96% (av. 85.6%) of the analysed populations were able to glide (one-way ANOVA with Tukey’s multiple comparisons test: HD100 vs D55A, p = 0.9947; HD100 vs D55E, p = 0.9791; D55A vs D55E, p = 0.9948). (**b**) The time taken for each strain to initiate gliding (onset of gliding) over the 6 hour time-lapse was assessed. The average onset of gliding of the site-directed mutant strains were found to not significantly differ (D55A = 2 hours; D55E = 1.9 hours) to the wild type gliding onset (HD100 = 2.2 hours) (one-way ANOVA with Tukey’s multiple comparisons test: HD100 vs D55A, p = 0.6536; HD100 vs D55E, p = 0.4299; D55A vs D55E, p = 0.9225). Results are from three biological replicates and two fields of view were analysed within each replicate.

Furthermore, both the site-directed mutant strains were still able to glide similarly to the wild type strain (Fig. 5). Within 6 hours of being applied to a 1% agarose surface, an average of 86.2% of the *B. bacteriovorus* RomR(D55A) population was able to glide, having an average onset of gliding motility of 2 hours (Fig. 5). Within this time frame, 86.8% of the RomR(D55E) population was able to glide, with an average gliding onset of 1.9 hours (Fig. 5). These data did not significantly differ to analyses of the wild type strain where 85.6% was of the population was able to glide, with a gliding onset of 2.2 hours (*p* = 0.99 and 0.98 for percentage of population gliding and *p* = 0.65 and 0.43 for onset of gliding by one way ANOVA; Fig. 5).

### Cell length differences observed between *B. bacteriovorus* RomR(D55A)-mCherry and wild type populations suggests a role for RomR_Bd_ in cell growth events

**Figure 6:**
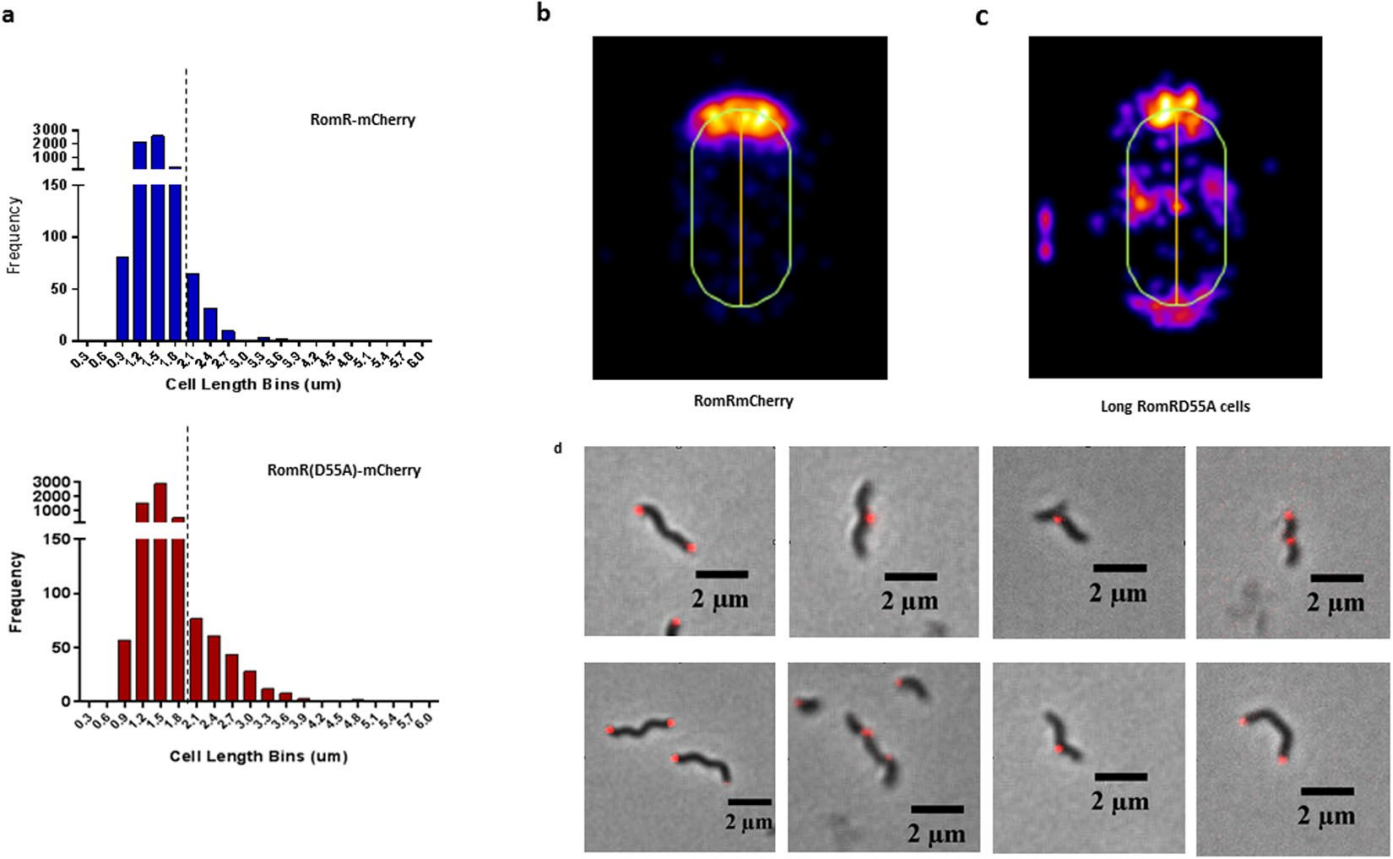
(**a**) Frequency distribution of cell lengths: *B. bacteriovorus* RomR-mCherry vs site-directed mutant RomR(D55A)-mCherry populations. The cells lengths of *B. bacteriovorus* populations expressing wild type mCherry tagged RomR [RomR-mCherry] and site-directed mutant RomR(D55A) [RomR(D55A)-mCherry] were assessed upon *B. bacteriovorus* completion of predatory replication, after bdelloplast exit (4 hours post infection – refer to Figs 2 and 4). Cell lengths were analysed from phase contrast microscopy images using MicrobeJ software^39^. Three biological repeats were carried out, with five fields of view analysed per strain for each replicate (n = 6119). Cell lengths were found to be significantly different between the populations using a Mann-Whitney test (*p* < 0.0001). (**b**) Cellular position maps of fluorescent foci detected by MicrobeJ software for RomRmCherry (n = 1863) in attack phase *B. bacteriovorus* HD100. RomRmCherry is almost exclusively found at one pole. (**c**) Cellular position maps of fluorescent foci detected by MicrobeJ software for RomRD55AmCherry for cells longer than 1.6 µm (n = 265). In these longer cells, there are often (34.7% of cells) multiple foci, and they are not all located at a single pole. (**d**) Merged fluorescent and phase contrast images of aberrant shaped long cells containing RomRD55AmCherry foci in varying locations in the cell. Scale bars are 2 µm.

Visible differences were noted in maximum cell lengths of populations comparing *B. bacteriovorus* RomR(D55A)-mCherry and RomR-mCherry strains, indicating that RomR_Bd_ is involved in the growth/division process, and that mutation of its putative phosphorylation site may somewhat affect the correct function of this protein, impacting *B. bacteriovorus* development (Fig. 6). We observed that there was a small, but significant, difference between the cell lengths of the two populations (Mann-Whitney test, *p* < 0.0001). The cell lengths observed in the wild type population ranged from 0.53–3.15 μm, with 2.1% of cells displaying lengths of 1.6 μm or above (Fig. 6). The D55A mutant population, however, displayed a significant increase in the frequency of longer cells compared to the wild type; lengths ranged from 0.53–4.46 μm, with 4.4% of cells measuring 1.6 μm or above (Fig. 6). The frequency of longer cells in the RomR(D55E)-mCherry mutant population were comparable to those in the wild type RomR-mCherry (Supplementary Fig. S2). As there is around a two-fold increase in the frequency of longer cells (1.6 μm and above) in the RomR_Bd_(D55A) mutant population, this suggests that aberrant division site placement during *B. bacteriovorus* growth could be occurring in some (but not all) of the bdelloplasts. These longer cells often (34.7%) had multiple fluorescent foci, either at both poles, or in the centre and the cells were bent or even branched, (Fig. 6d) suggesting that the specification of their poles was aberrant. Although these results do not propose a strong role for RomR_Bd_ phosphorylation at D55, they do imply that the protein is involved in *B. bacteriovorus* cell growth/septal placement events. It is likely that replacing the acidic aspartate residue at position 55 in RomR_Bd_ with the small, uncharged alanine residue, has partially interfered with the correct functioning of this protein in some circumstances during intracellular growth of *B. bacteriovorus*.

### Informatic co-evolution predictions of potential histidine-kinase partners for REC protein, RomR_Bd_, yielded candidate proteins tested for binding

Deletion of RomR_Bd_ in our previous study was not possible, indicating that RomR_Bd_ is essential^5^. The tolerated D55A/E site-directed mutations of RomR_Bd_ suggest that phosphorylation is not essential for viability and growth throughout the predatory cycle (with the exception of minor effects on possible septal positioning observed with the D55A mutation). This implies that the *B. bacteriovorus* RomR_Bd_ system may have diverged from the proteobacterial ancestral situation to no longer require phosphorylation for its role. As *romR*_*Bd*_ is in a single gene operon, it encodes an orphan response regulator, lacking an obvious cognate kinase meaning that either it does not have one, or that the cognate kinase is encoded elsewhere in the genome. Recently, a number of studies have identified the specificity determining residues of kinases and their cognate receiver domains^18–20^. This informatics approach predicts how contacting residues may have co-evolved in REC domains and their cognate kinases^21,22^. There are seven positions in the receiver domain that largely determine which kinases the receiver domain will interact with^18^. We examined these specificity-determining residues for RomR_Bd_ and found that five of the seven residues matched those found in REC containing protein CheY1 from *Rhodobacter sphaeroides*. CheY1 is phosphorylated by three different CheA kinase proteins from *R. sphaeroides*^23–25^, and we therefore reasoned that any of the CheA homologues (Bd0578, Bd2406 and Bd3469) found in *B. bacteriovorus* HD100 could be the cognate kinase for RomR_Bd_. Bacterial two-hybrid (B2H) analysis was employed to assess whether RomR interacts with any of these sensor kinases; this has been used previously to determine interactions between sensor kinase and response regulator pairs^26^.

**Figure 7:**
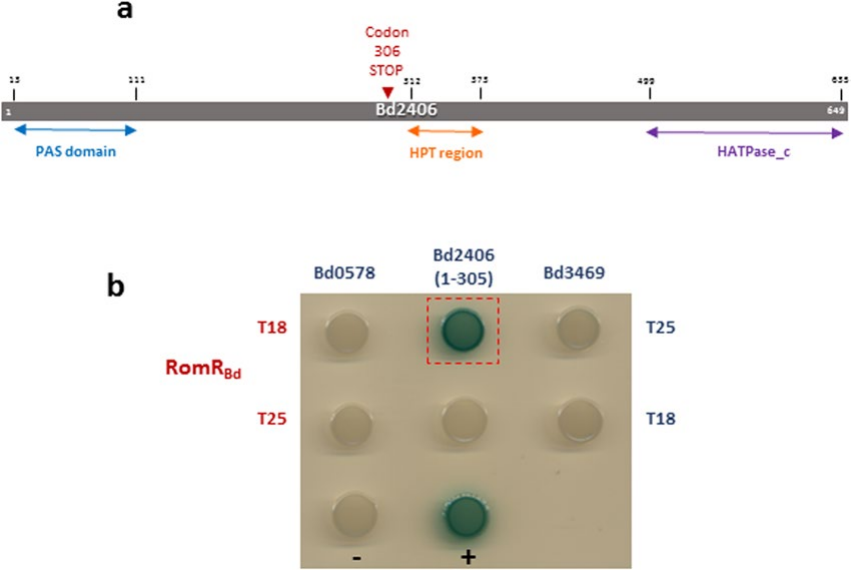
(**a**) Diagrammatic representation of unfolded, genome-sequence predicted, full length Bd2406 and the domains within the protein. The *B. bacteriovorus* HD100 protein is only translated up to amino acid 305 due to a mutation of a lysine at position 306 to a stop codon within the encoding gene. This mutation lies directly upstream of the histidine phosphotransfer (HPT) region of the protein (red arrow). Therefore, the translated portion of this protein in HD100 contains only the sensory region of this kinase (the PAS domain). At the C-terminus is the ATPase region of the kinase (HATPase_c). (**b**) Spot plates (on LB containing kanamycin, ampicillin and X-gal) after 48 hours of incubation at 29 °C summarising bacterial two-hybrid study results carried out to investigate interactions between *B. bacteriovorus* RomR (RomR_Bd_) and predicted cognate kinases. Proteins were tagged at their N-termini with either the T25 or T18 fragment of adenylate cyclase and interactions between protein pairs assayed for in *E. coli* BTH101. Formation of a blue colony suggests an interaction between RomR_Bd_ and amino acids 1–305 of Bd2406 [Bd2406(1–305)].

However, upon further analysis of the genes encoding these candidate sensor kinases, gene *bd2406* was found to contain a difference from the published *B. bacteriovorus* HD100 genome sequence at codon 306, modifying the original lysine codon (AAA) to a stop codon (TAA) (Fig. 7). This stop mutation was not seen in the originally published HD100 genome sequence^14^, which was acquired from low coverage, Sanger sequencing and may have given rise to errors, hence why the mutation was not picked up until now (see methods for genome entry amendments). The codon difference resides just upstream of the histidine phosphotransfer (HPT) domain of Bd2406 (Fig. 7a). This was intriguing, as when potential interactions between the *B. bacteriovorus* HD100 RomR_Bd_ protein and each of these three kinases were assessed via bacterial two hybrid (B2H) analysis in *E. coli*, the N-terminal remnant region of Bd2406 (amino acids 1–305) was found to interact with RomR_Bd_, whereas the other potential kinases (Bd0578 and Bd3469) did not interact with RomR_Bd_ (Fig. 7b). Bd2406 amino acids 1–305 contain a PAS sensory domain but lack the histidine phosphotransfer and HATPase domains. The ancestral protein Bd2406 was likely to have been a sensor kinase, but it exists now in *B. bacteriovorus* HD100 as a truncated product that retains the ability to interact with RomR_Bd_ in the B2H assay.

**Figure 8:**
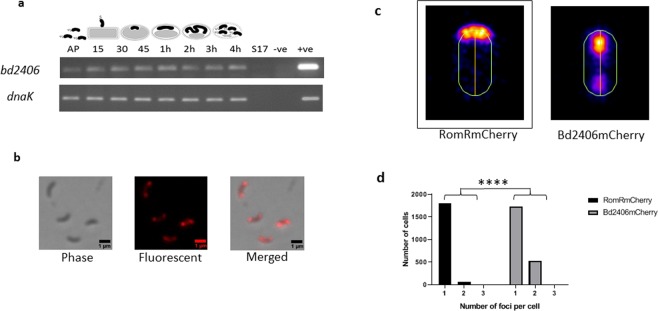
(**a**) Expression pattern of *B. bacteriovorus* HD100 HIS-kinase related gene, *bd2406*, and control gene *dnaK*, throughout the predatory life cycle using RT-PCR with transcript specific primers. RNA was prepared from identical volumes of infection cultures with *B. bacteriovorus* HD100 predator and *E. coli* S17-1 prey at time-points throughout the predatory life cycle represented by the cartoon above. AP: RNA from attack-phase *B. bacteriovorus*, 15–45: RNA from 15–45 minute time-points through the predatory cycle, 1–4 h: RNA from 1–4 hours through the time-course, S17: *E. coli* S17-1 only template RNA, −ve: no template negative control, + ve: *B. bacteriovorus* HD100 genomic DNA template as positive control. (Full length agarose gel is presented in supplementary fig. S9). (**b**) Epifluorescence phase contrast microscopy displaying the localisation of naturally truncated Bd2406(1–305) protein tagged C-terminally with mCherry in *B. bacteriovorus* attack-phase cells. Scale bars are 1 µm. (**c**) Cellular position maps of fluorescent foci detected by MicrobeJ software for RomRmCherry (n = 1863; this map [black box] is also displayed in Fig. 6) and Bd2406mCherry (n = 2258) in attack phase *B. bacteriovorus* HD100. RomRmCherry is almost exclusively found at one pole, while Bd2406mCherry (N-terminus) is found at both subpole locations. (**d**) Histogram depicting the number of fluorescent foci of RomRmCherry and Bd2406mCherry per cell. Bd2406mCherry had significantly (*p* < 0.0001 by Mann-Whitney test) differing numbers of detectable foci compared to RomRmCherry.

RNA-seq analysis from *B. bacteriovorus* host-independent strain, HID13 (originally derived from HD100) suggests that a nearly complete transcript of *bd2406* is made (transcription appears to terminate ~99 bp before the end of the gene), with a sharp decrease in transcription being observed immediately after the premature stop codon 306 (Supplementary Fig. S3)^27^. RT-PCR, with a set of primers annealing to the 5′ of the gene (which encodes the N-terminal PAS domains of the protein), also established that this transcript is present throughout the predatory life cycle of *B. bacteriovorus* HD100 (Fig. 8a). Additionally, fluorescent localisation studies in *B. bacteriovorus* expressing Bd2406(1–305)-mCherry demonstrated that amino acids 1–305 of this protein are still translated and this region of the protein displays a predominantly subpolar localisation (Fig. 8b–d), often with two foci (23.3% of cells with detectable foci), significantly (*p* < 0.0001 by Mann-Whitney test) more than was observed for RomR-mCherry (3.6% of cells containing 2 detectable foci). Apart from the premature stop codon, the *bd2406* gene is otherwise still intact within the strain HD100; mutation and loss of Bd2406 kinase function may be a relatively recent evolutionary event.

Homologues of *bd2406* are present within all other sequenced *B. bacteriovorus* invasive-strain genomes (Supplementary Fig. S4). The sequence of *bd2406* homologues from strains 109J (closely related to HD100 cultured for 50 years in labs, originally isolated from soil)^28^ and Tiberius (freshly isolated in 2010 from a river)^29^, were investigated. The full-length *bd2406* gene coding sequence, without an amino acid 306 stop codon, was detected within both the 109J and Tiberius genomes. These strains have been isolated from varied ecosystems (soil/water) and are therefore subject to different selective pressures, which may be why strain HD100 contains a now truncated *bd2406* gene compared to the other strains. It is also possible that strains have different ways of regulating their poles; for example, strain Tiberius is unusual as 1% of the population naturally grows host-independently in both a budding and septating manner^29^.

These data imply that RomR_Bd_ has diverged from an ancestral deltaproteobacterial sequence to no longer require phosphorylation for its current role in predatory replication of *B. bacteriovorus* HD100. We suggest that the gene encoding Bd2406 may have descended from the ancestral RomR_Bd_-cognate-kinase gene, existing as a (still expressed) truncated gene within the strain HD100 genome. Although the kinase region of Bd2406 is not translated (only the N-terminal PAS domain), we still detected an interaction between the N-terminal remnant of Bd2406 and RomR_Bd,_ from strain HD100, in the B2H analysis, suggesting that these proteins may still interact *in vivo*.

A *B. bacteriovorus bd2406* deletion strain was generated and found to progress through the predatory life cycle similarly to how the wild type strain, HD100, carries out predatory replication^30,31^ (Supplementary Fig. S5). Moreover, RomR-mCherry is monopolar in the *bd2406* deletion strain in attack-phase (Supplementary Fig. S6), similarly to when localisation is observed in wild type *B. bacteriovorus* HD100 (Fig. 2). These data therefore further indicate that Bd2406 (including the translated truncated portion from residues 1–305) no longer has a function linked to dynamic regulation of RomR in *B. bacteriovorus*.

### The actual *B. bacteriovorus* HD100 RomR_Bd_ translation start site begins after key receiver domain residues present in the *M. xanthus* homologue

BLASTP analysis revealed RomR_Bd_ homologues to be present in all sequenced *B. bacteriovorus* strains with the closely related prey-invasive strains to HD100: 109 J and Tiberius^28,29^, showing genome annotations with 99% and 97% identity to the HD100 encoded protein respectively. The less related strains [ArHS^32^, W, R0, BER2, EC13 – Genbank unpublished] show 66–74% identities with the HD100 protein (Supplementary Fig. S7).

When *romR*_*Bd*_ sequences from the genomes of more recently isolated *B. bacteriovorus* strains (Tiberius, RO, EC13 BER2, ArHS) were examined (Supplementary Fig. S7), the REC domain-coding regions were found to be present in the DNA but not contained within the predicted RomR protein open reading frame. These genes also seemed to be truncated at the 5′ end and this caused us to question whether the *B. bacteriovorus* HD100 RomR_Bd_ sequence might be as the original annotation had predicted. If annotated incorrectly, then the transcriptional, and therefore translational, start of RomR is actually further downstream within the *romR* gene sequence (corresponding to coding triplet 20 of the originally annotated HD100 *romR* sequence). This would mean that D and E residues for initial stages of phosphoryl group acceptance in a potential REC domain (D_11_E_12_ equivalent in HD100 RomR) may not be coded for in the amino acids of the gene product (Supplementary Fig. S7). As a consequence, phosphorylation of RomR_Bd_ may not actually take place. With these genomic data in mind, we investigated whether the REC domain is correctly annotated within the HD100 gene product.

RNA-seq (from *B. bacteriovorus* host-independent strain, HID13 - originally derived from HD100^27^) was interrogated to probe the transcriptional start of RomR_Bd_ from *B. bacteriovorus* HD100 and therefore any potential translational start sites downstream of it. The transcript was observed to begin around 7 bps into the originally predicted *romR*_*Bd*_ protein coding sequence (Supplementary Fig. S8). This suggests the true RomR_Bd_ start site is the truncated one predicted in the *B. bacteriovorus* Tiberius genome sequence, which resides downstream of the originally predicted HD100 translational start (codon 20) (Supplementary Figs S7 and S8). If correct, then this excludes the residues, D_11_E_12_, normally essential for REC domain function.

**Figure 9:**
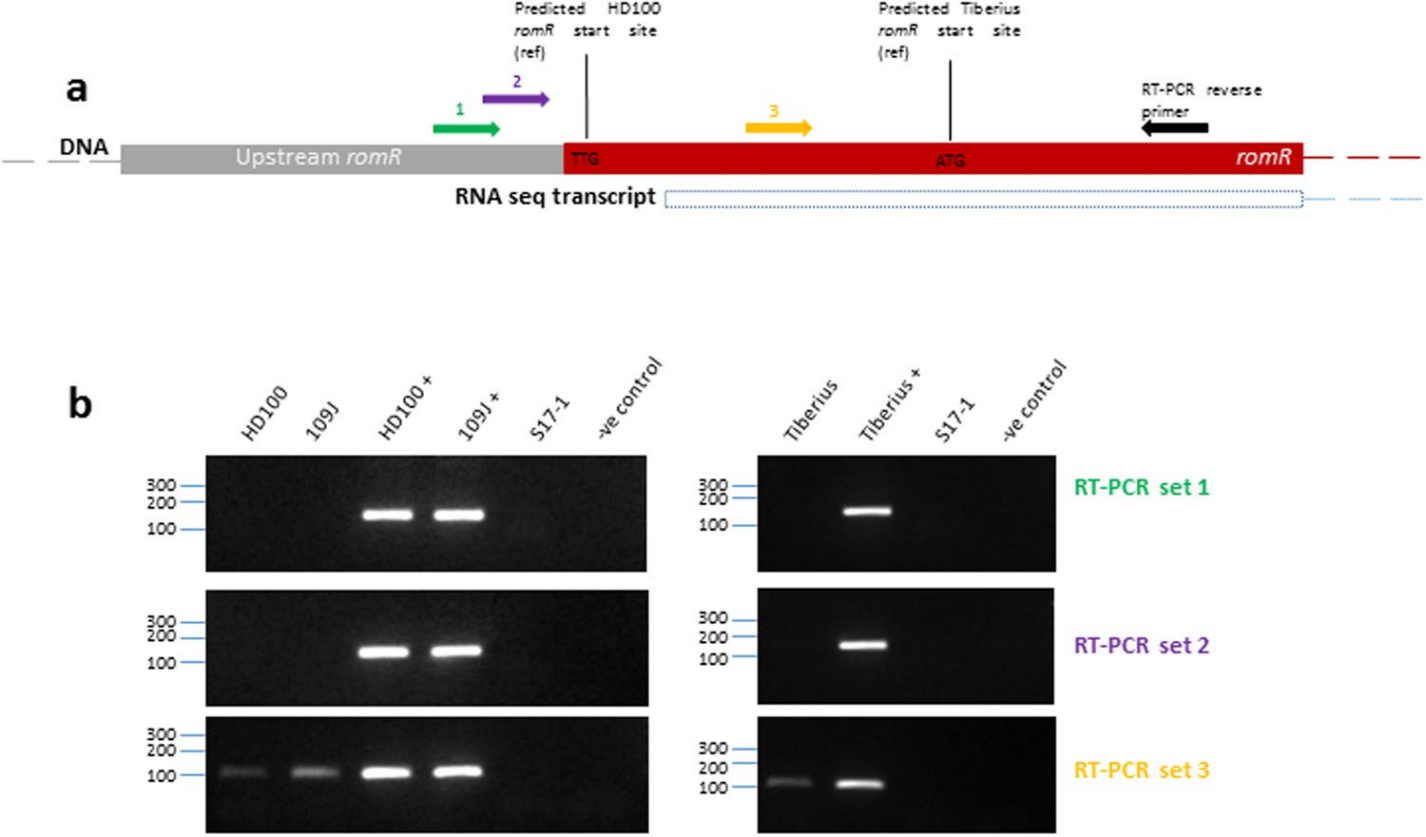
(**a**) Diagrammatic representation of the *B. bacteriovorus* HD100 *romR* gene and upstream region, demonstrating the start of the RNA transcript determined by RNA seq data for HD100 (Supplementary Fig. S8). The transcript starts ~7 bps into where the *B. bacteriovorus* HD100 amino acid sequence was predicted to begin by original genome annotation^14^. RT-PCR primer binding sites for RT-PCR (in part B) are displayed (forward primers for RT-PCR sets 1, 2 and 3 and the reverse primer used in all reactions). (**b**) RT-PCR was carried out to confirm the *B. bacteriovorus* HD100 *romR* transcript start site. RT-PCR sets used different forward primers (1, 2 & 3) which bound either upstream of the predicted gene start (sets 1 and 2) or downstream of the predicted transcription start site suggested from RNA seq data (set 3) (Supplementary Fig. S8). Forward primers 1, 2 & 3 are depicted in [A.], and all were used in conjunction with the reverse primer (a different reverse primer was used for the Tiberius assays due to slight sequence variation; this binds to the same place on the transcript as the HD100 and 109 J reverse primer). Full length gels are presented in Supplementary Figs S11–S13. HD100, 109 J, Tiberius: RNA samples extracted from attack-phase *B. bacteriovorus* HD100, 109J and Tiberius cells. HD100+ , 109 J+ and Tiberius+ : genomic DNA template from the corresponding wild type strains used for positive controls. S17-1: *E. coli* S17-1 only template RNA control. −ve: no template negative control. A control set of RT-PCRs were also carried out which used primers to *B. bacteriovorus dnaK*, giving around a 100 bp product in all RNA and genomic DNA samples.

To confirm this, RT-PCR was carried out on total RNA extracted from *B. bacteriovorus* HD100, 109J and Tiberius attack-phase cells, with primers annealing just upstream and downstream of the transcript start site observed in the RNA-seq data (Fig. 9a)^27^. The RT-PCR product observed in each case demonstrates the likely *romR*_*Bd*_ translational start codon is the one originally predicted in the *B. bacteriovorus* Tiberius genome annotation (Fig. 9b)^29^ which was formerly annotated as M_20_ in the MQLAL stretch of the HD100 RomR_Bd_ sequence (Supplementary Fig. S7).

Finding the RomR REC domain truncation in *B. bacteriovorus* strain HD100 leads us to conclude that phosphorylation does not play a part in the regulation/role of this protein. This truncation makes any ancestral cognate kinase redundant, and may be the reason for selection of the mutation observed in the *bd2406* gene of strain HD100.

## Discussion

A homologue of motility control protein RomR_Mx_ which oscillates between the cell poles to establish polarity required for gliding motility reversals in the wolf pack predator *M. xanthus*^9–11^ has evolved an alternative function in the intraperiplasmic lone predatory bacterium, *B. bacteriovorus*.

The *B. bacteriovorus* RomR_Bd_ is monopolar at the non-flagellar, piliated pole, in attack-phase cells^5^ and begins to diffuse slowly to the opposite cell pole (between 30 mins–1 hour timepoints during the replicative predatory life cycle) following prey invasion, eventually displaying a predominantly bipolar localisation between 45 mins–2 hour timepoints (Fig. 2a,b) as the predator grows within the dead prey cell. At 3 hours, multiple foci were observed within the bdelloplast containing the developing *Bdellovibrio* filament (Fig. 2a,b). This number correlates, at one focus per *Bdellovibrio* pole, with the known progeny number of *B. bacteriovorus* released by a single prey bdelloplast, which is 3–4^31^. This cellular expression pattern correlates with RomR_Bd_ having an essential role during the growth of *B. bacteriovorus*, which was proposed by Milner *et al*.^5^ who found that obtaining cells with a full *romR*_*Bd*_ deletion was not possible. Thus, RomR_Bd_ may have an essential role in determining and/or establishing poles and regulating growth of the intraperiplasmic *Bdellovibrio* from these poles.

We determined that during *B. bacteriovorus* gliding motility outside prey on a solid surface, RomR_Bd_ is present at the predominantly leading cell pole, and does not change position during cell motility reversals. This contrasted to previous published findings in *M. xanthus* where fluorescently tagged RomR_Mx_ clusters at the lagging cell pole with the model that it dynamically relocalises upon cell motility reversals, migrating quickly (within 30 seconds) to the opposite pole to create a new lagging pole pre-empting the MglA movement and the reversals^9^. This has, however, been contradicted in *M. xanthus* by recent findings by Guzzo *et al*.^12^ which still infer a dynamic role for RomR_Mx_ movement, but at a much slower pace of 160 seconds to traverse a cell length. Despite this, the difference is that there is no relocalisation of RomR_Bd_ during gliding motility.

Cell-motility reversals, on solid surfaces, differ between the two predators. *B. bacteriovorus* gliding reversals are extremely brief; being more like a chemotactic tumble required for generation of a random directional change. This compares to *M. xanthus* where cells are able to move in the opposite direction for extended periods of time; resetting polarity with dynamic relocalisations of RomR/MglA/MglB proteins seeming to be essential in *Myxococcus* for this to occur. This correlates with *B. bacteriovorus* having a single predatory (piliated) pole rather than pili alternating at both poles, as in *Myxococcus*. The refractory period set by RomR_Mx_ signalling proposed by Guzzo *et al*.^12^ allows for extended cell movement in the opposite direction, in contrast to the brief directional changes observed in *B. bacteriovorus*. GTP-bound MglA_Mx_ and RomR_Mx_ have been found to interact in *M. xanthus*, enabling them to work together with other components of the Frz system and polarity proteins to allow for that extended bidirectional movement^10,11^. No interaction was seen between RomR_Bd_ and MglA_Bd_ (which is in a permanent GTP bound state) in previous bacterial two-hybrid studies^5^. Both proteins do however interact with a tetratricopeptide repeat (TPR) protein, which similarly localises to the predatory pole^5^. It is possible that this protein links MglA_Bd_/RomR_Bd_ to their different functions in *B. bacteriovorus*, keeping them monopolar (in attack-phase). Alternatively, this may be a relevant ancestral tripartite interaction that is moderated by Frz protein interactions in *Myxococcus*.

We also demonstrate that site-directed mutagenesis of RomR_Bd_ at its putative phosphorylation site (D55A) has no effect on RomR_Bd_ localisation in attack-phase *B. bacteriovorus* or throughout the predatory life cycle, suggesting this protein is not phosphorylated or that a phosphorelay is not required for RomR_Bd_ function throughout predation. Dissimilarly, mutation of the predicted phosphorylation site of RomR_Mx_ was initially reported (although these initial findings are also further discussed and challenged by Guzzo *et al*.^12^) to show significant effects on *M. xanthus* behaviour^9^. An increased frequency of motility reversals were witnessed with the phosphomimic phosphorylation site mutant, RomR_Mx_(D53E), whereas no reversals were observed when the unphosphorylatable protein, RomR_Mx_(D53N), was present^9^. We found no differences in *B. bacteriovorus* gliding motility when the corresponding D55 RomR_Bd_ amino acid was mutated (to either an alanine or a glutamate residue).

There was a subtle increase in the frequency of aberrant, longer cell morphologies in the *B. bacteriovorus* population expressing the RomR_Bd_ D55A site-directed mutant protein. Here, the number of cells measuring greater than 1.6 µm more than doubled in the mutant population compared to the wild type (4.4% of the mutant population compared to 2.1% of the wild type population). This suggests that the amino acid at this site only has a minor effect on RomR_Bd_ function and that this is only affecting cell septation/growth-control in a fraction of *B. bacteriovorus* predatory progeny. Linked to this, RomR_Bd_ displays bipolar localisation during bdelloplast growth, (when *Bdellovibrio* are stationary but elongating inside prey cells), and increased transcription of *romR*_*Bd*_ could be detected between 1–3 hours of prey invasion (coinciding with predator growth inside bdelloplast), which again suggests a developmental function. Involvement in polar growth events fits with the observation that a viable *romR*_*Bd*_ deletion mutant could not be achieved by Milner *et al*.^5^ but that D55 point-mutated RomR_Bd_ proteins are viable. However, whether RomR_Bd_ as a growth-controller is a scaffold protein that recruits other proteins to the poles, or has a direct role in growth, is currently unknown. Harvey and coworkers^33^ established that RomR_Mx_ is recruited to the site of *M. xanthus* cell division to reset polarity for the daughter cells. This allows asymmetric localisation, important for motility in the parent cell, to be emulated in the daughter cells^33^. They suggested that RomR_Mx_ may interact with proteins of the developing cell poles; it is therefore conceivable that RomR_Bd_ works together with polar growth components in *B. bacteriovorus*, having evolved a key role in establishing polarity during cell division^33^.

Correlating with our initial finding that the predicted RomR_Bd_ REC domain phosphorylation site (D55) is no longer key to protein function, a full-length cognate kinase was not identified for RomR_Bd_. The predictive kinase:response regulator bioinformatic interaction studies carried out here did, however, yield the candidate *bd2406*, with a stop codon residing in the coding sequence just upstream of the kinase region in the genome of strain HD100. RomR_Bd_ was surprisingly found to interact using bacterial two-hybrid analysis with the remaining N-terminal translated region of Bd2406, which includes a sensory PAS domain. Further work here has demonstrated that RomR_Bd_ actually has a truncated REC domain and so the essential acid pocket residues are omitted from the REC domain. This almost certainly renders the REC domain non-functional, providing further evidence that phosphotransfer does not take place in this system, but that the current-truncated interacting RomR_Bd_-Bd2406 protein pair in strain HD100 has evolved from an ancient, full-length sensor kinase system.

Generally across bacteria, in the absence of their cognate response regulator, there is potential for unwanted cross-talk between kinases and non-cognate response regulators^34,35^. Therefore, the evolution of an early stop codon in *bd2406* (if the ancestral RomR_Bd_ cognate kinase), removing this kinase activity, could help to avoid this unwanted cross-talk. The RomR_Bd_/Bd2406 interactions that we observed in the bacterial two-hybrid assay involved only the N-terminus of the Bd2406 (prior to the stop codon that removes the kinase domains from the HD100 protein) indicating RomR_Bd_ interacts with the PAS-domain-containing residual portion of Bd2406 but this did not impact RomR_Bd_ localization or predatory replication (observed in a *bd2406* deletion strain).

Although point mutational evidence suggests the *M. xanthus* RomR_Mx_ protein is phosphorylated^9^, recent work by Guzzo *et al*.^12^ proposes that this is not actually the case and RomR_Mx_ remains unmodified for its function. This now poses interesting evolutionary questions as to when *M. xanthus* and *B. bacteriovorus* diverged and when phosphorylation was lost (it could indeed have been before the divergence of these genera). The genes encoding the MglA (MglB) signalling system and the associated RomR protein shown here have evolved to have a different function in *B. bacteriovorus*. This is supported by Milner and co-workers, who demonstrated a prey-invasion phenotype, type IV pilus associated role for MglA (and the absence of MglB)^5^. Our discoveries here that fluorescently tagged RomR_Bd_ is still expressed and changes cellular address during intrabacterial growth but not gliding motility, and that mutating the predicted phosphorylation site (which is tolerated without lethality) has very minor effects on cell length, suggests that RomR_Bd_ does interact with key proteins for intrabacterial cell division. This complements previous findings by Milner and coworkers where the entire *romR*_*Bd*_ gene could not be deleted^5^.

Defining mechanisms of RomR_Bd_ regulation of such intra-bacterial growth processes for *B. bacteriovorus* are complex, as many evolutionarily modified cell division proteins exist in *Bdellovibrio* and they require extensive study beyond the scope of this work. However, our studies here demonstrate that evolutionary modifications, from an original deltaproteobacterial paradigm, in *B. bacteriovorus* have shaped its function as a lone intrabacterial predator with novel pole specification and cell division regulation.

## Methods

### Bacterial strains, Plasmids and growth conditions

Bacterial strains and plasmids used in this work are listed in supplementary Table S1. Host-dependent *Bdellovibrio bacteriovorus* strains were cultured on *Escherichia coli* S17-1 cells in Ca-HEPES buffer and incubated at 29 °C^2,36^. Primers used to construct these plasmids and strains are shown in Supplementary Table S2.

### Generation of *B. bacteriovorus* deletion and site-directed mutants

The markerless *bd2406* deletion was generated by a method previously described^37^, adapted from a method by Steyert & Pineiro^38^. Spliced overlap extension PCR was used to join 1 kb flanking regions either side of the gene of interest; the DNA product was then inserted into the suicide vector pK18*mobsacB* and conjugated into *B. bacteriovorus* HD100 using the conjugative donor strain *E. coli* S17-1. The resulting merodiploid *B. bacteriovorus* strain, where the plasmid was integrated into the genome via a single cross-over in the flanking regions of the *bd2406* gene, was then grown in the presence of sucrose to induce a second recombination event to remove the target gene, leaving only the flanking regions.

The *romR* site-directed mutant strains (D55A/D55E) were generated using a similar method. The mutant *romR* sequences were generated by overlap-extension PCR (with around 1 kb of flanking sequence either side of the gene) and inserted into the sucrose suicide vector pK18*mobsacB*. The protocol was then carried out as for a markerless deletion to fully replace the wild type gene in the genome with the mutant version.

### Fluorescent Protein Tagging

The *B. bacteriovorus* RomR-mCherry strain used in this study was previously created by Milner and co-workers via conjugation of the vector, pK18_*romR*-mCherry in *B. bacteriovorus* HD100^5^. Here, a PCR method based on the QuickChange^TM^ site-directed mutant protocol developed by Stratagene (La Jolla, CA) used pK18_*romR*-mCherry as the template to generate vectors to tag the site-directed mutant *romR* genes (D55A/D55E) at their 3′ ends with the mCherry gene. The vectors were conjugated (via *E. coli* S17-1) into the corresponding *B. bacteriovorus romR* site-directed mutant strains. Resulting *B. bacteriovorus* merodiploid strains (plasmid integrated by single cross-over event at the *romR* gene within the genome) were selected for with kanamycin.

### Phase-contrast and Fluorescence Microscopy

Fluorescence, Phase-contrast and time-lapse microscopy (for mCherry tagged protein localisation, cell length and gliding motility studies) was carried out on either a Nikon Eclipse E600 Epifluorescence microscope or a Nikon Ti-E inverted fluorescence microscope.

### Equipment and Settings

The Nikon Eclipse E600 epifluorescence microscope was equipped with a 100x objective lens, hcRED filter where necessary (excitation 560 to 600 nm; emission 610 to 665 nm) and a Hamamatsu Orca ER camera. Images were analysed on Simple PCI software version 6.6.

The Nikon Ti-E microscope with was equipped with a Plan Apo 60x /1.40 100x Oil Ph3 DM objective, 1.5x intermediate magnification, a Cy5 filter cube and an Andor Neo sCMOS camera, using the mCherry settings for detection of mCherry tagged proteins (emission maximum 610–660 nm) where necessary.

### Image analysis

Images were enhanced using the sharpen and smooth tools of ImageJ and SimplePCI software.

Cell lengths were analysed via ImageJ software (MicrobeJ plug-in)^39^. *B. bacteriovorus* cells were defined on MicrobeJ using the following parameters: area 0–1.5 μm^2^, length 0.5–5 μm, width 0.2–0.8 μm, and all other parameters were set as default. The image was then manually inspected to confirm that the majority of the cells had been correctly identified. To analyse the long cells containing RomRD55AmCherry, the length was defined as 1.6–5 µm.

Fluorescent foci were detected using default maxima settings with the foci method and associations with parent bacteria had a tolerance of 0.1 µm.

### Host-dependent *B. bacteriovorus* Invasion Assays

To observe the localisation of *B. bacteriovorus* mCherry tagged proteins during predatory growth within *E. coli* prey cells, a 50 ml culture of the required *B. bacteriovorus* strain (merodiploid), at 2.5 × 10^8^ cells per ml, was concentrated 10-fold and combined with 4 ml *E. coli* S17-1 containing pZMR100 (for kanamycin resistance) at OD_600_ 1 in CaHEPES and 3 ml CaHEPES to begin the *B. bacteriovorus* predatory cycle. Cell suspensions were incubated at 29 °C with 200 rpm. Progress through the predatory cycle (and position of the mCherry tagged protein) was visualised via fluorescence microscopy (one second exposure) at time-points: 0, 15, 30, 45 mins and then 1, 2, 3 and 4 hours, by taking 10 μl of the cell mixture and immobilising on 1% agarose slide.

### Gliding motility studies

*B. bacteriovorus* gliding motility was assessed via time-lapse microscopy on the Nikon Eclipse E600 epifluorescence microscope. *B. bacteriovorus* cells from 1 ml of predatory culture, at 2.5 × 10^8^ cells per ml, were concentrated 10-fold in CaHEPES and applied to a 1% agarose:CaHEPES surface on a microscope slide (containing wells to allow hydration). Time-lapse microscopy was run over the course of 6 hours with 150 second intervals between frames.

Fluorescent gliding studies (0.8 second exposure) were carried out to determine the localisation of mCherry tagged proteins within *B. bacteriovorus* during gliding motility. The strain of interest was applied to a 1% agarose surface (as above) and incubated for 2 hours at room temperature prior to time-lapse microscopy (to allow gliding initiation). Fluorescent time-lapse microscopy was then carried on the Nikon Eclipse E600 epifluorescence microscope out over 20 minutes with 150 second intervals between frames.

Analyses on the resulting videos were carried out in ImageJ using the cell counter tool to determine onset of gliding and percentage of cells gliding within a population. Two biological replicates (and two fields of view within each replicate) were analysed. To record RomRmCherry localisation during gliding, 1668 cells were observed and of these, 149 cell reversals were observed with the fluorescent signal still bright enough to determine the location. For the other cells, either the cells did not reverse during the recorded time, or the fluorescent signal had bleached such that its location could not be clearly elucidated.

### RNA

Synchronous *Bdellovibrio bacteriovorus* HD100 predatory infections on *E. coli* S17-1 (as well as S17-1 control alone) were set up as previously described^2^. Samples were taken throughout the timecourse and total RNA isolated using a Promega SV total RNA isolation kit. RNA quality was tested using an Agilent Bioanalyser using the RNA Nano kit.

RT-PCR was carried out using the Qiagen One-step RT-PCR kit under the reaction conditions: one cycle 50 °C for 30 mins, 95 °C for 15 mins, then 25 cycles of 94 °C for 1 min, 48 °C for 1 min, 72 °C for 2 mins, and finally a 10 mins extension at 72 °C. Primers are listed in Table S2 and experiments were carried out with at least two biological replicates. Transcript analysis for *bd2406* and *bd2761 (romR)* was carried out as previously described^27^.

### Kinase Prediction for RomR

Following procedures from the systematic evolutionary studies of sensor-kinase to response-regulator co-evolution by the Laub lab^18,20,21^, the specificity determining residues of RomR were identified by aligning the protein sequence of RomR, using Clustal Omega, with the receiver domains of the four well-studied kinases where the specificity residues have been determined (OmpR, RstA, CpxR from *E. coli* and RR468 from *T. maritima*). In this alignment, the residues aligning with OmpR D13, R15, L16, L20, R22, Y23, N108 are the specificity residues^18^. For RomR the residues at these positions are: STIVLAD. These bear a striking resemblance to the specificity residues of CheY1 from *R. sphaeroides* (STILEAD), which matches five of these seven residues. As CheY1 is phosphorylated by three different CheA proteins from *R. sphaeroides*, we hypothesised that RomR may be phosphorylated by one or more of the CheA homologues from *B. bacteriovorus* (Bd0578, Bd2406 and Bd3469) and interactions were tested for by 2 hybrid analysis.

### Bacterial Two-Hybrid Analysis

Bacterial two-hybrid analysis of interacting protein partners was carried out using the Euromedex system^40^. Each gene was cloned into both pUT18C and pKT25 bacterial two-hybrid vectors to tag the corresponding protein N-terminally with either the T18 or T25 domain of adenylate cyclase (CyaA). Protein interactions were investigated by co-transforming plasmids (one pUT18C and one pKT25 – supplementary Table S1) containing the encoding genes into the *E. coli cyaA*^*−*^ strain, BTH101. Transformations were plated onto nutrient agar containing kanamycin (25 μg/ml), ampicillin (50 μg/ml) and X-gal (40 μg/ml) and incubated at 29 °C for 48 hours. A colour change is observed in this system when protein interactions occur, with *E. coli* colonies turning blue on the media. When the proteins interact the two fragments of CyaA are brought together, which increases the cellular cyclic-AMP levels, triggering the transcriptional activation of catabolic operons, in this case *lac* operon^40^. Co-transformants were grown in LB medium overnight and then spotted onto nutrient agar plates (+Amp +Kan +X-gal) and incubated again for 48 hours to assess any interactions. The reciprocal test for each interaction was also carried out, along with positive and negative controls (Euromedex). Analyses were carried out in triplicate, with three biological repeats.

### Statistical analyses

To assess whether the differences observed between cell lengths of the *B. bacteriovorus* populations were significant, a D’Agostino-Pearson omnibus or Shapiro-Wilks (for samples with fewer datapoints) normality tests were first used to evaluate whether the data was normally distributed. Where data was determined to have a normal distribution, then one-way ANOVA was used, where normality tests failed,the non-parametric Mann-Whitney test was applied to assess the significance between the data sets. These tests were carried out using GraphPad Prism.

### Bioinformatic tools

Protein BLAST searches were carried out using the ncbi tool (https://blast.ncbi.nlm.nih.gov) and the pFAM (http://pfam.xfam.org/) and ncbi conserved domain (www.ncbi.nlm-nih.gov/Structure/cdd/wrpsb.cgi) tools were utilised for protein domain analyses. Protein alignments were generated using CLUSTALW (http://embnet.vital-it.ch/software/ClustalW.html) and displayed using BoxShade (http://embnet.vital-it.ch/software/BOX_form.html).

### DNA Sequencing

Sanger sequencing was performed by Source Bioscience www.sourcebioscience.com).

### Accession Numbers

The updated *B. bacteriovorus* HD100 *bd2761 (romR)* and *bd2406* gene sequences were deposited in genbank and new accession numbers are: bd2761_ORF_B.bacteriovorus_HD100 - MK113826, and bd2406_truncated_B.bacteriovorus_HD100 - MK113827.

## Supplementary information

Supplementary data and information

## Data Availability

The data that support the findings of this study are available from the corresponding author upon request.
