# Supplementary material for: Evolutionary diversification of the RomR protein of the invasive deltaproteobacterium, Bdellovibrio bacteriovorus
Source: Sci Rep. 2019 Mar 21;9:5007. doi: 10.1038/s41598-019-41263-5 (PMC6428892; doi:10.1038/s41598-019-41263-5)
Supplement: Supplementary file 1 — Supplementary data and information [file 41598_2019_41263_MOESM1_ESM.docx]

**Supplementary material for manuscript entitled:**

**Evolutionary diversification of the RomR protein of the invasive deltaproteobacterium, *Bdellovibrio* *bacteriovorus***

**Authors:** Rebecca C Lowry, David S Milner, Asmaa SM Al-Bayati, Carey Lambert, Vanessa I Francis, Steven L Porter, RE Sockett

**Supplementary Tables**

| Strain | Genotype/description and use | Reference/  Source |
| --- | --- | --- |
| ***Escherichia coli* strains*:*** |  |  |
| S17-1 | *thi,pro,hsdR*^-^,*hsdM*^+^,*rec*A; integrated plasmid RP4-Tc::Mu-Km::Tn*7*; used as donor for conjugating plasmids into *Bdellovibrio* | (1) |
| S17-1 pZMR100 | S17-1 containing pZMR100 vector; used as prey for Km^r^ *Bdellovibrio* strains |  |
| BTH101 | F-, *cya-99*, *araD139, galE15, galK16, rpsL1 (Str r)*, *hsdR2, mcrA1, mcrB1* | Euromedex |
| ***Bdellovibrio* strains*:*** |  |  |
| *B. bacteriovorus* HD100 | Wild type *B. bacteriovorus* host-dependent strain | (2) |
| *B. bacteriovorus* RomR-mCherry | HD100 with single-crossover of pK18::*romR*-mCherry; *romR* ORF fused to *mCherry* expressed from native promoter, and promoterless copy of *romR* | (3) |
| *B. bacteriovorus romR(D55A)* | HD100 with wild type *romR* replaced with site-directed mutant, *romR(D55A)* | This study |
| *B. bacteriovorus romR(D55E)* | HD100 with wild type *romR* replaced with site-directed mutant, *romR(D55E)* | This study |
| *B. bacteriovorus* RomR(D55A)-mCherry | *B. bacteriovorus* *romR(D55A)* containing a single-crossover of pK18::*romR(D55A)*-mCherry *romR(D55A)* ORF fused to *mCherry* expressed from native promoter, and promoterless copy of *romR(D55A)* | This study |
| *B. bacteriovorus* RomR(D55E)-mCherry | *B. bacteriovorus* *romR(D55E)* containing a single-crossover of pK18::*romR(D55E)*-mCherry *romR(D55E)* ORF fused to *mCherry* expressed from native promoter, and promoterless copy of *romR(D55E)* | This study |
| **Plasmids:** |  |  |
| pK18*mobsacB* | Km^r^ sucrose suicide vector used for conjugation and recombination into *Bdellovibrio* genome | (4) |
| pK18_*romR-mCherry* | pK18*mobsacB* containing *romR* (*bd2761*) ORF with C-terminal mCherry tag. | (3) |
| pK18_*romR(D55A)* | pK18*mobsacB* containing *romR(D55A)* and around 1 kb of flanking genomic sequence either side of the gene, for creating site-directed *B. bacteriovorus* strain in HD100 | This study |
| pK18_*romR(D55E)* | pK18*mobsacB* containing *romR(D55E)* and around 1 kb of flanking genomic sequence either side of the gene, for creating site-directed *B. bacteriovorus* strain in HD100 | This study |
| pK18_*romR(D55A)-mCherry* | pK18*mobsacB* containing *romR* (*bd2761*) ORF with the site-directed mutation D55A and a C-terminal mCherry tag | This study |
| pK18_*romR(D55E)-mCherry* | pK18*mobsacB* containing *romR* (*bd2761*) ORF with the site-directed mutation D55E and a C-terminal mCherry tag | This study |
| pZMR100 | λ defective vector, Km^r^. Used to confer Km^r^ for S17-1 used as prey | (5) |
| pUT18C | Amp^r^ bacterial two-hybrid vector, for fusion of chosen gene to 3’ of *cyaA*(T18) fragment (creates N-terminally tagged protein of choice) | Euromedex |
| pKT25 | Km^r^ bacterial two-hybrid vector, for fusion of chosen gene to 3’ of *cyaA*(T25) fragment (creates N-terminally tagged protein of choice) | Euromedex |
| pUT18C_*bd0578* | pUT18C containing *bd0578* ORF | This study |
| pKT25_*bd0578* | pKT25 containing *bd0578* ORF | This study |
| pUT18C_*bd2406(1-305)* | pUT18C containing *bd2406(codons1-306)* ORF | This study |
| pKT25_*bd2406(1-305)* | pKT25 containing *bd2406(codons1-306)* ORF | This study |
| pUT18C_*bd3469* | pUT18C containing *bd3469* ORF | This study |
| pKT25_*bd3469* | pKT25 containing *bd3469* ORF | This study |
| pUT18-zip control | pUT18C containing the leucine zipper region from yeast GCN4 | Euromedex |
| pKT25-zip control | pKT25 containing the leucine zipper region from yeast GCN4 | Euromedex |

**Supplementary Table S1**. Strains and Plasmids

| Primer | Sequence | Description |
| --- | --- | --- |
| ***Cloning Primers*** |  |  |
|  |  |  |
| 2761_upstream(F) | GCGC**GGATCC**AATAGGCGAAAACCAGGGC | Froward primer to amplify from ~1 kb upstream of *bd2761*, contains **BamHI** site, to create mutated *bd2761* fragments either with mutational reverse primers or 2761_downstream(R) |
| 2761_downstream(R) | GCGC**GGATCC**AAATGCCATGGCACCG | Reverse primer to amplify from ~200 kb downstream of *bd2761*, contains **BamHI** site, to create mutated *bd2761* fragments either with mutational forward primers or 2761_upstream(F) |
| 2761_D55A(F) | GTGTTCGCC**GCT**GTGTTGTT | Site-directed mutational forward primer, with mutated codon 55 (encoding **D55A**) at the centre |
| 2761_D55A(R) | AACAACAC**AGC**GGCGAACAC | Site-directed mutational reverse primer, with mutated codon 55 (encoding **D55A**) at the centre |
| 2761_D55E(F) | GTGTTCGCC**GAG**GTGTTGTT | Site-directed mutational forward primer, with mutated codon 55 (encoding **D55E**) at the centre |
| 2761_D55E(R) | AACAACAC**CTC**GGCGAACAC | Site-directed mutational reverse primer, with mutated codon 55 (encoding **D55E**) at the centre |
| 2406del_upF | GCGC**TCTAGA**AAACCGCCAGCAACAATG | Forward primer to amplify ~1 kb upstream of the *bd2406* gene to create the deletion construct pK18_Δ*bd2406*, contains an **XbaI** site for ligation into pK18*mobsacB* |
| 2406del_upR | **TCGGTGATGTAA**GTCTCATTTTACGA | Reverse primer to amplify ~1 kb upstream of the *bd2406* gene to create the deletion construct pK18_Δ*bd2406.* |
| 2406del_downF | **TCGTAAAATGAGA**CTTACATCACCGA | Forward primer to amplify ~1 kb downstream of the *bd2406* gene to create the deletion construct pK18_Δ*bd2406* |
| 2406del_downR | GCGC**TCTAGA**CCGGGGATGGCCAT | Reverse primer to amplify ~1 kb downstream of the *bd2406* gene to create the deletion construct pK18_Δ*bd2406*, contains an **XbaI** site for ligation into pK18*mobsacB* |
|  |  |  |
| ***RT Primers*** |  |  |
| **2761_RT_F** | **CTCAGGATGACGAGCTTTCC** | Forward internal primer used to detect *bd2761* transcription by RT-PCR |
| **2761_RT_R** | **GGTGCTTTGGTTTTGGTCAC** | Reverse internal primer used to detect *bd2761* transcription by RT-PCR |
| **2761_RTupF1** | GACTGGCGGCGGAAATC | Forward RT-PCR primer binding 11-27 bps upstream of the originally predicted *bd2761* translational start site in HD100 (2). For investigations into transcriptional start site of the *B. bacteriovorus* HD100 *bd2761* gene and 109J/Tiberius homologues. |
| **2761_RTupF2** | GCGGAAATCCAATTTAAC | Forward RT-PCR primer binding 2-19 bps upstream of the originally predicted *bd2761* translational start site in HD100 (2). For investigations into transcriptional start site of the *B. bacteriovorus* HD100 *bd2761* gene and 109J/Tiberius homologues. |
| **2761_RTdownF** | GTCTTGCTTGCAGATGAGAG | Forward RT-PCR primer binding 19-38 bps downstream of the originally predicted *bd2761 translational* start site in HD100 (2). For investigations into transcriptional start site of the *B. bacteriovorus* HD100 *bd2761* gene and 109J/Tiberius homologues. |
| **2761_RTstartR** | GCAATACATCAAGGCCCAC | Reverse RT-PCR primer for investigations into transcriptional start site of the *B. bacteriovorus* HD100 *bd2761* gene and the 109J homologue. |
| **TibRomR_RTstartR** | GCAATACGTCAAGACCCAC | Reverse RT-PCR primer for investigations into transcriptional start site of the *B. bacteriovorus* Tiberius *romR (bd2761* from HD100) homologue. |
| 2406_RT_F1 | TGATTCCATGGGTGACAAGA | Forward internal primer used to detect *bd2406* transcription by RT-PCR upstream of the mutation at codon 306 |
| 2406_RT_R1 | GTTCAGCGCGGTATTTCTTC | Reverse internal primer used to detect *bd2406* transcription by RT-PCR upstream of the mutation at codon 306 |
| 2406_RT_F2 | GGTATGGACGCCATCAAAGT | Forward internal primer used to detect *bd2406* transcription by RT-PCR downstream of the mutation at codon 306 |
| 2406_RT_R2 | TCGGTGATGTAAGGGACCTC | Reverse internal primer used to detect *bd2406* transcription by RT-PCR downstream of the mutation at codon 306 |
| dnaK_RT_F | TGAGGACGAGATCAAACGTG | Forward internal primer used to detect *B. bacteriovorus* HD100 *dnaK* transcription by RT-PCR |
| dnaK_RT_R | AAACCAGGTTGTCGAGGTTG | Reverse internal primer used to detect  *B. bacteriovorus* HD100 *dnaK* transcription by RT-PCR |

**Supplementary Table S2. Primers -** Site-directed mutational forward and reverse primer pairs in Table S2 were not only used to generate the required *bd2761* fragments to create the site-directed mutant *B. bacteriovorus* strains, but also to amplify site-directed mutant pK18::*romR*-mCherry constructs (Table S1) via an adapted QuickChange^TM^ site-directed mutagenesis PCR method (originally developed by Stratagene - La Jolla, CA)

|  | replicate |  |  |  |  |  |  |
| --- | --- | --- | --- | --- | --- | --- | --- |
| timepoint (mins) | | 15 | 30 | 45 | 60 | 120 | 180 |
| RomRmCherry | 1 | 16 | 58 | 42 | 37 | 36 | 22 |
| RomRmCherry | 2 | 13 | 53 | 49 | 57 | 58 | 39 |
| RomRmCherry | 3 | 48 | 37 | 22 | 26 | 53 | 46 |
| RomRD55AmCherry | 1 | 74 | 39 | 50 | 52 | 53 | 37 |
| RomRD55AmCherry | 2 | 21 | 50 | 32 | 48 | 50 | 39 |
| RomRD55AmCherry | 3 | 33 | 28 | 34 | 46 | 35 | 44 |
| RomRD55EmCherry | 1 | 44 | 50 | 51 | 39 | 51 | 54 |
| RomRD55EmCherry | 2 | 57 | 45 | 27 | 42 | 84 | 47 |
| RomRD55EmCherry | 3 | 25 | 35 | 46 | 63 | 56 | 55 |

**Supplementary Table S3-** n values for number of bdelloplasts analysed and scored for fluorescent foci reported in Figures 2 and 4.


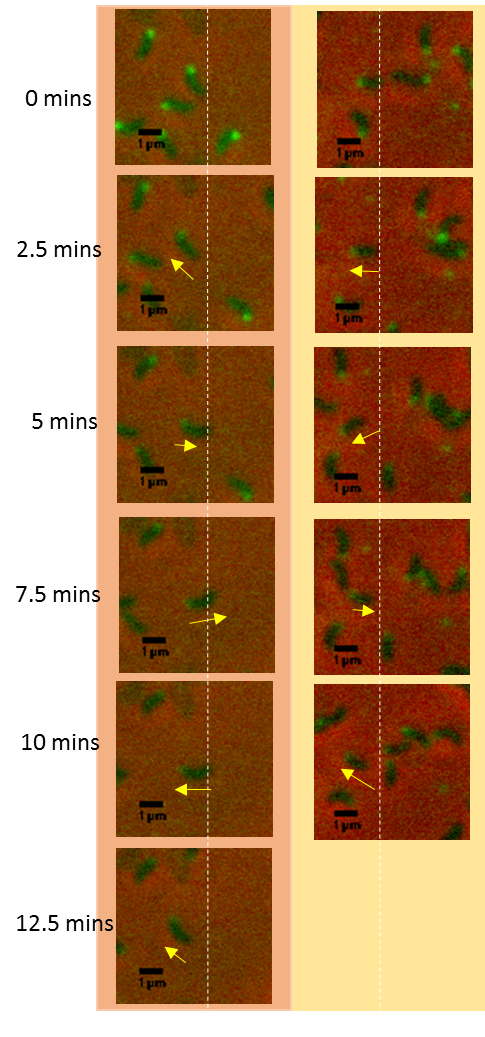


**Supplementary Figure S1** Further examples of epifluorescence time-lapse microscopy over a 12.5 minute period displaying constant localisation of RomR-mCherry during *B. bacteriovorus* gliding motility. The strain, *B. bacteriovorus* RomR-mCherry, was incubated on a 1% agarose surface for 2 hours prior to time-lapse microscopy in order to allow gliding motility to initiate. The RomR-mCherry focus stayed at one pole regardless of direction of motility. Yellow arrow shows direction of motility. Images are representative of 149 cell reversals observed in two independent experiments.

**
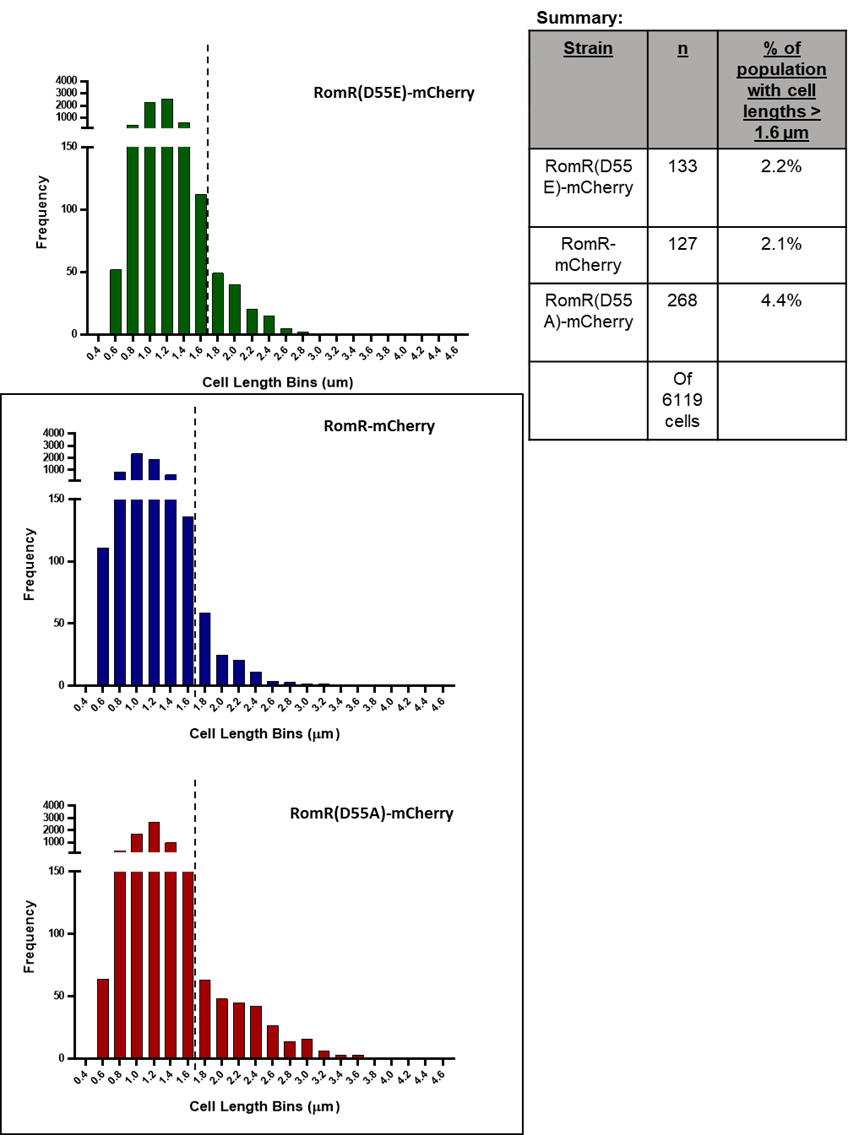
Supplementary Figures**

**Supplementary Figure S2.** The boxed data is from figure 6 in the manuscript displaying the frequency distribution of cell lengths in *B. bacteriovorus* populations expressing wild type mCherry tagged RomR [RomR-mCherry] and the tagged site-directed mutant RomR(D55A) [RomR(D55A)-mCherry]. We also show the spread of cell lengths observed in the *B. bacteriovorus* population expressing the RomR(D55E) site directed mutant tagged with mCherry [RomR(D55E)-mCherry]. Cell lengths were assessed upon *B. bacteriovorus* completion of predatory replication, after bdelloplast exit (4 hours post infection – please refer to Figures 3 & 4 of the manuscript). Cell lengths were analysed from phase contrast microscopy images using MicrobeJ software (6). Three biological repeats were carried out, with five fields of view analysed per strain for each replicate (n = 6119).

The spread of cell lengths in the RomR(D55E)-mCherry population were more comparable to those observed in the wildtype (RomR-mCherry) population with the percentage of cells measuring 1.6 μm or above accounting for 2.2% of the population (similarly to 2.1% of the RomR-mCherry population), compared to the RomR(D55A) population where there is a two-fold increase in the occurrence of the longer cells (4.4% of the population).

**
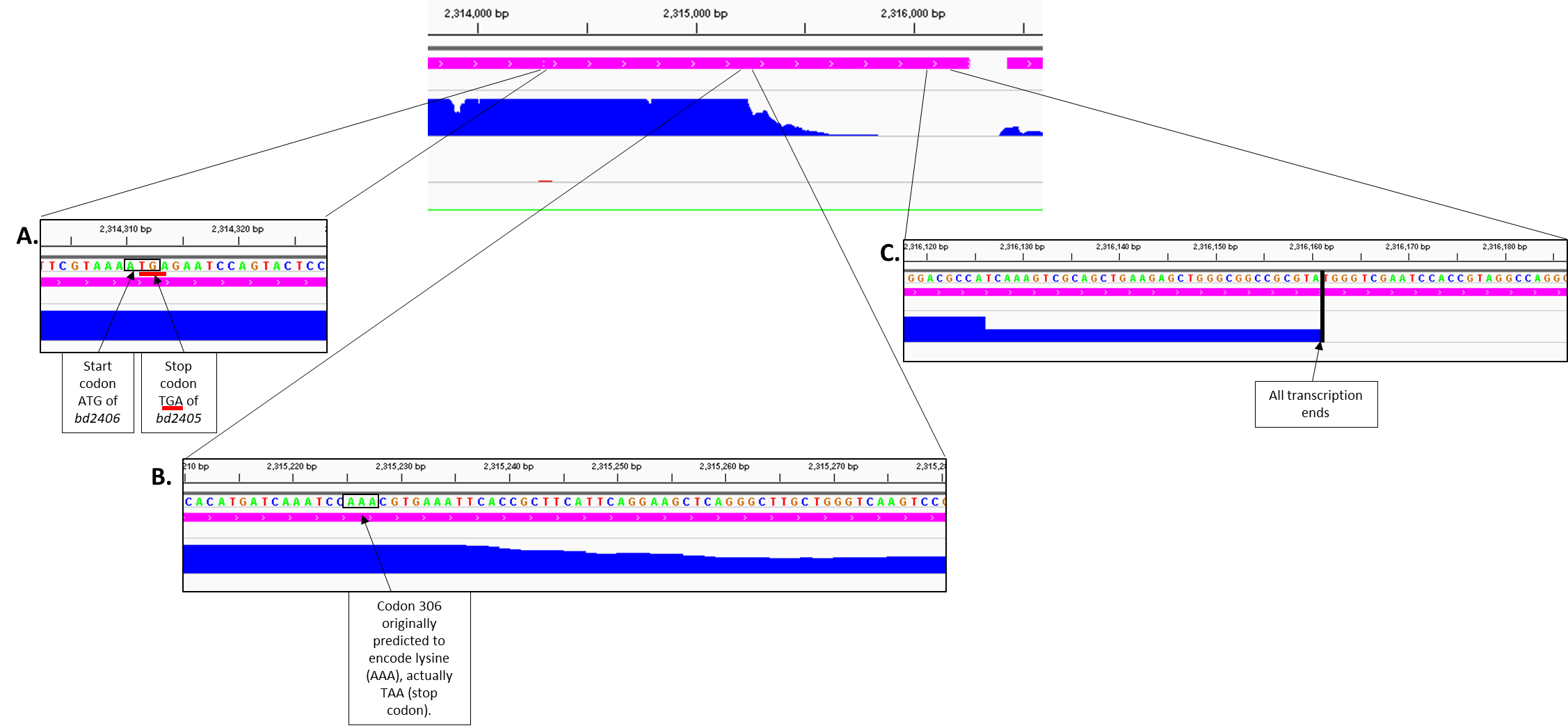
**

**Supplementary Figure S3.** RNA-seq data showing the transcript of *bd2406* from *B. bacteriovorus* HID13 (HI derivative of strain HD100; SRA accession PRJNA505601)(7). RNA-seq reads are mapped onto the genome sequence of *B. bacteriovorus* HD100 (2) and shown as a bar chart in blue. 0-703 reads were mapped to the region shown, which has been autoscaled for ease of viewing. The start of the *bd2406* gene can be observed in **A**. Codon 306 (originally predicted to encode a lysine reside – AAA – but is actually present as a stop codon – TAA – in the HD100 genome) can be seen in **B**. The end of the transcript can be seen more clearly in **C**. A sharp decrease in transcription can be observed after the natural mutation at codon 306. Here, RNA-seq data suggests the transcription of this gene terminates at the sequence ‘CGGCCGCGTA’ which is 99 bps before the end of the originally predicted, full-length gene. It is possible that the stop codon results in the degradation of the normally longer transcripts, with less protection from RNA polymerase complex, hence why the majority of transcripts are much shorter than the length of the predicted, uninterrupted gene.

**
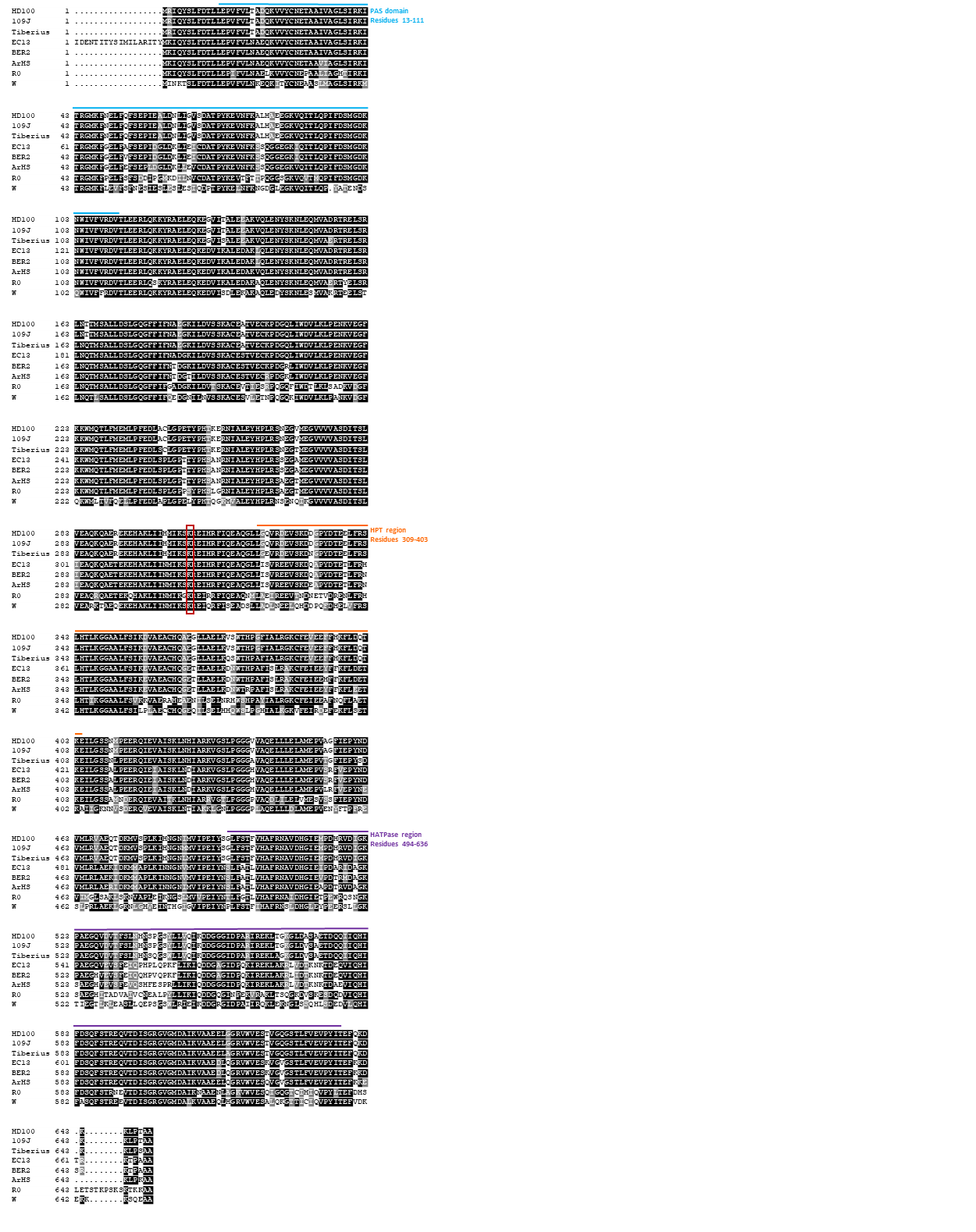
**

**Supplementary Figure S4**. Alignment of the primary amino acid sequence of *B. bacteriovorus* HD100 Bd2406 with homologues from other *B. bacteriovorus* strains: 109J (99%), Tiberius (97%), EC13 (82%), BER2 (82%), ArHS (81%), R0 (70%) and W (64%). The percentages in brackets show the identity these homologues share with the HD100 protein. Domains are defined above the HD100 Bd2406 sequence (ncbi conserved domain) and the lysine reside mutated in the HD100 sequence to a stop codon (K306) is highlighted. The alignment was carried out using CLUSTALW and displayed with BoxShade.


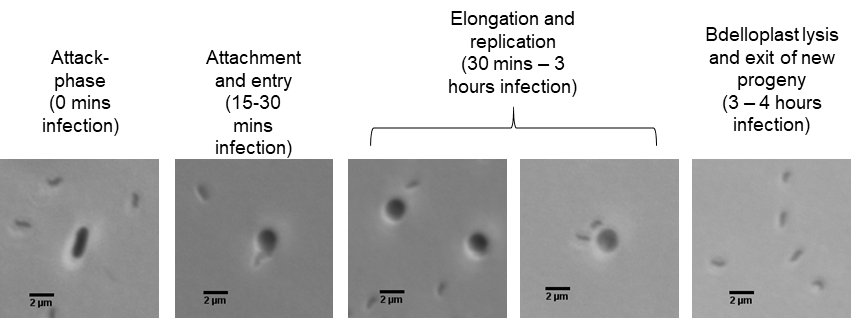


**Supplementary Figure S5.** Phase-contrast microscopy demonstrating the predatory ability of the *B. bacteriovorus bd2406* deletion strain over the course of 4 hours using *E. coli* S17-1 prey.


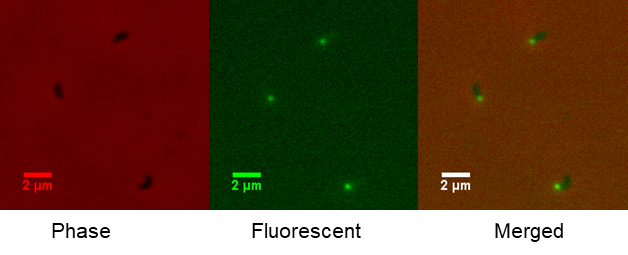


**Supplementary Figure S6.** Epifluorescence phase-contrast microscopy demonstrating the localisation of RomR_Bd_-mCherry within the *B. bacteriovorus bd2406* deletion strain in attack-phase.


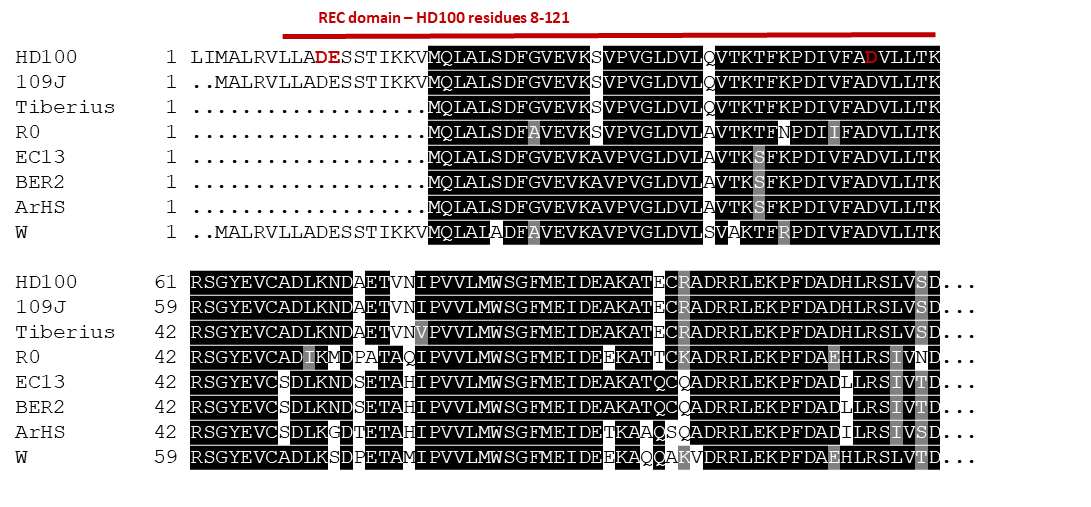


**Supplementary Figure S7**. Alignment of the primary amino acid sequence of the RomR_BdHD100_ receiver domain (residues 8-121 [ncbi conserved domain]) (2) with RomR homologues from *B. bacteriovorus* strains: 109J (8), Tiberius (9), ArHS (10), R0 (genbank unpub.), BER2 (genbank unpub.) and W (genbank unpub.). Highlighted region is the predicted sequence of the HD100 RomR protein from original genome annotation.

**
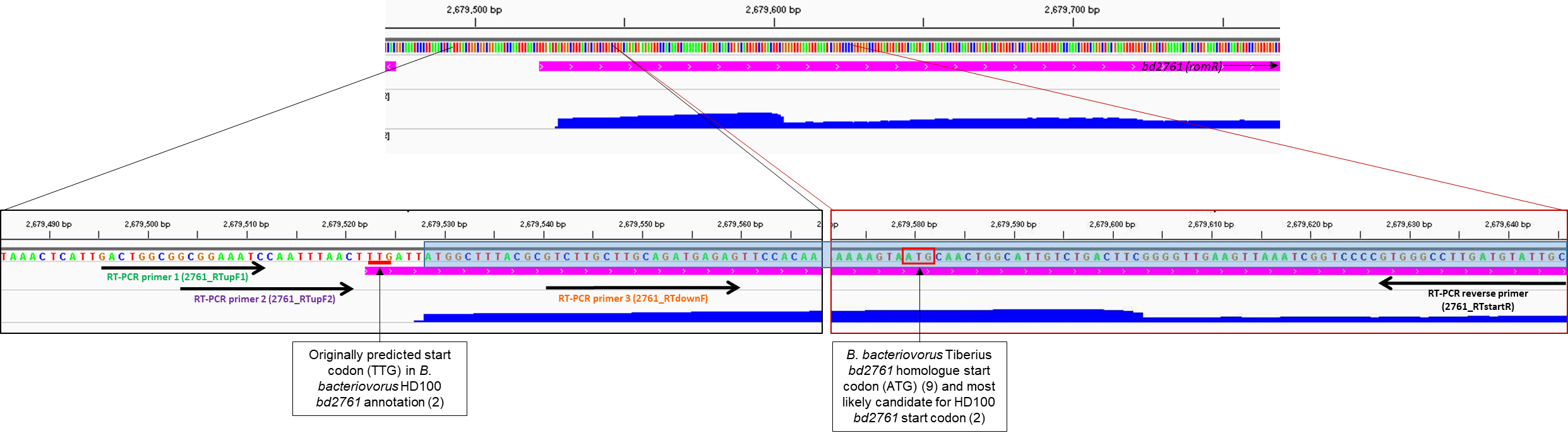
**

**Supplementary Figure S8.** RNA-seq data showing the transcript of *bd2761* (*romR*) from *B. bacteriovorus* HID13 (HI derivative of strain HD100; SRA accession PRJNA505601) (7). RNA-seq reads are mapped onto the genome sequence of *B. bacteriovorus* HD100 (2) and are shown as a bar chart in blue. 0-119 reads were mapped to the region shown, which has been autoscaled for ease of viewing. These data demonstrate that transcription of *bd2761* begins ~7 bps into the original sequence annotation of *bd2761 (2)*. The correct translational start site of *bd2761* from strain HD100 is, therefore, downstream of this. The most likely candidate (ATG) is highlighted (which is also predicted to be the start site of the *bd2761* homologue from strain Tiberius)(9).

Binding sites of primers used in transcriptional start site investigations from Figure 9 are also shown.

**Full agarose gel images**


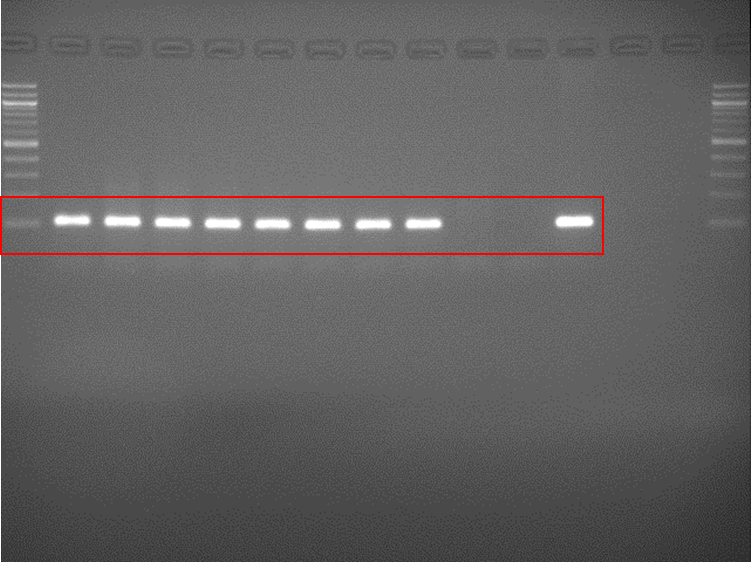


300

100

200


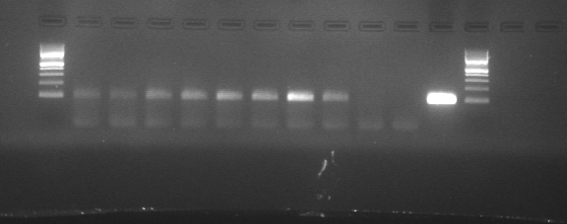


100

200

300


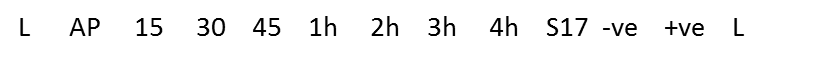


Supplementary figure S9 - Agarose gel (2%) corresponding to RT-PCR (*romRBd* and *dnaK* transcription throughout the *B. bacteriovorus* HD100 host-dependent life-cycle) shown in Figure 2 of the manuscript. Cropped region highlighted. The samples were run with a NEB 100bp ladder.


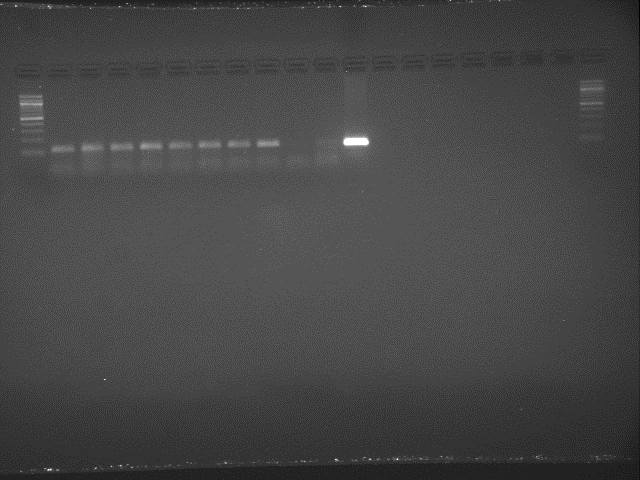

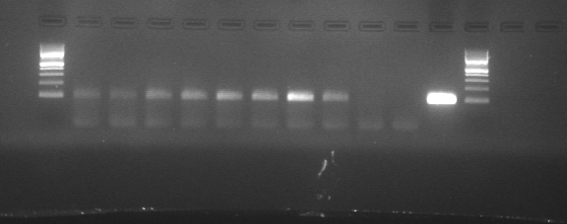


300

200

100

Supplementary figure S10 - Agarose gel (2%) corresponding to RT-PCR (*bd2406* transcription throughout the *B. bactervorus* HD100 host-dependent life-cycle) shown in Figure 8 of the manuscript. Cropped region highlighted. The samples were run with a NEB 100bp ladder.

100

300

200

300

200

100

Supplementary Figure S11 - Agarose gel (2%) corresponding to *B. bacteriovorus* HD100 and 109J RT-PCR results shown in Figure 9 of the manuscript. Cropped region highlighted. The samples were run with a NEB 100bp ladder.


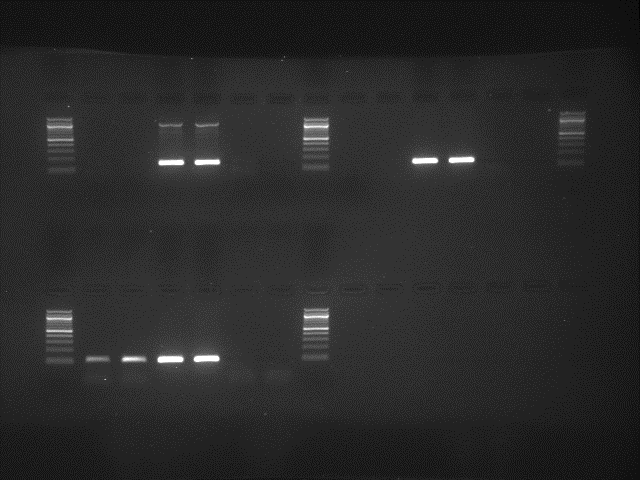


**RT-PCR set 2**

**RT-PCR set 3**

**RT-PCR set 1**

300

200

100


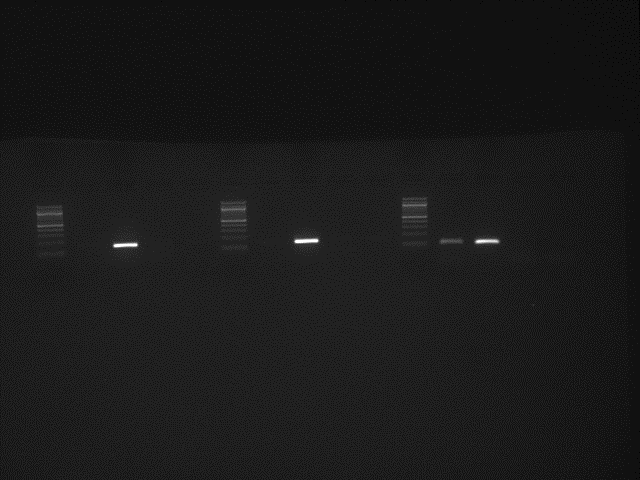


**RT-PCR set 1**

**RT-PCR set 2**

**RT-PCR set 3**

Supplementary figure S12 - Agarose gel (2%) corresponding to *B. bacteriovorus* Tiberius RT-PCR results shown in Figure 9 of the manuscript. Cropped region highlighted. The samples were run with a NEB 100bp ladder.

300

200

100

Supplementary figure S13 - Agarose gel (2%) corresponding to *B. bacteriovorus dnaK* control RT-PCR results shown in Figure 9 of the manuscript. Cropped region highlighted. The samples were run with a NEB 100bp ladder.


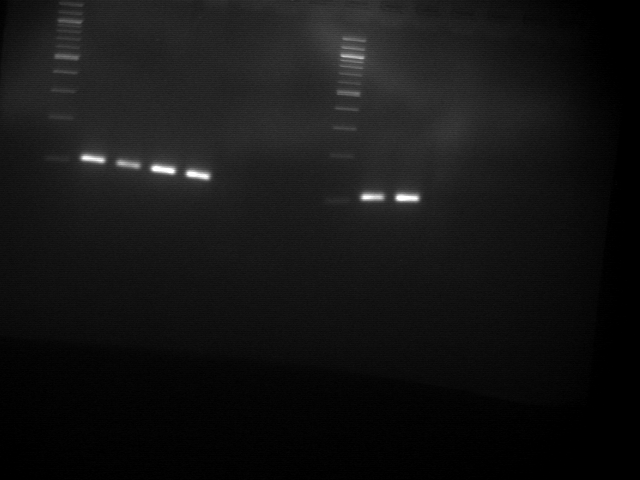


**Supplementary references**

1. Simon R, Preifer U, Puhler A. A broad host range mobilisation system for *in vivo* genetic engineering: transposon mutagenesis in gram negative bacteria. Biotechnology. 1983;9:184-91.

2. Rendulic S, Jagtap P, Rosinus A, Eppinger M, Baar C, Lanz C, et al. A predator unmasked: life cycle of *Bdellovibrio bacteriovorus* from a genomic perspective. Science. 2004;303(5658):689-92.

3. Milner DS, Till R, Cadby I, Lovering AL, Basford SM, Saxon EB, et al. Ras GTPase-Like Protein MglA, a Controller of Bacterial Social-Motility in Myxobacteria, Has Evolved to Control Bacterial Predation by *Bdellovibrio.* PLoS Genet. 2014;10(4):e1004253.

4. Evans KJ, Lambert C, Sockett RE. Predation by *Bdellovibrio bacteriovorus* HD100 requires type IV pili. Journal of bacteriology. 2007;189(13):4850-9.

5. Rogers M, Ekaterinaki N, Nimmo E, Sherratt D. Analysis of Tn7 transposition. Molecular and General Genetics. 1986;205(3):550-6.

6. Ducret A, Quardokus EM, Brun YV. MicrobeJ, a tool for high throughput bacterial cell detection and quantitative analysis. Nature microbiology. 2016;1(7):16077-.

7. Capeness MJ, Lambert C, Lovering AL, Till R, Uchida K, Chaudhuri R, et al. Activity of *Bdellovibrio hit* locus proteins, Bd0108 and Bd0109, links Type IVa pilus extrusion/retraction status to prey-independent growth signalling. PLoS One. 2013;8(11):e79759.

8. Wurtzel O, Dori-Bachash M, Pietrokovski S, Jurkevitch E, Sorek R. Mutation detection with next-generation resequencing through a mediator genome. PLoS One. 2010;5(12):e15628.

9. Hobley L, Lerner TR, Williams LE, Lambert C, Till R, Milner DS, et al. Genome analysis of a simultaneously predatory and prey-independent, novel *Bdellovibrio bacteriovorus* from the River Tiber, supports in silico predictions of both ancient and recent lateral gene transfer from diverse bacteria. BMC Genomics. 2012;13:670-.

10. Sangwan N, Lambert C, Sharma A, Gupta V, Khurana P, Khurana JP, et al. Arsenic rich Himalayan hot spring metagenomics reveal genetically novel predator–prey genotypes. Environmental Microbiology Reports. 2015;7(6):812-23.
